# Impact of doxycycline post-exposure prophylaxis for sexually transmitted infections on the gut microbiome and antimicrobial resistome

**DOI:** 10.1038/s41591-024-03274-2

**Published:** 2024-10-03

**Authors:** Victoria T. Chu, Abigail Glascock, Deborah Donnell, Cole Grabow, Clare E. Brown, Ryan Ward, Christina Love, Katrina L. Kalantar, Stephanie E. Cohen, Chase Cannon, Michael H. Woodworth, Colleen F. Kelley, Connie Celum, Anne F. Luetkemeyer, Charles R. Langelier

**Affiliations:** 1 Department of Pediatrics, Division of Infectious Diseases and Global Health, University of California, San Francisco, San Francisco, CA USA; 2 Chan Zuckerberg Biohub, San Francisco, CA USA; 3 Fred Hutchinson Cancer Center, Seattle, WA USA; 4 Department of Global Health, University of Washington, Seattle, WA USA; 5 Department of Medicine, Division of Infectious Diseases, University of California, San Francisco, San Francisco, CA USA; 6 Chan Zuckerberg Initiative, Redwood City, CA USA; 7 San Francisco Department of Public Health, San Francisco, CA USA; 8 Department of Medicine, University of Washington, Seattle, WA USA; 9 Department of Medicine, Division of Infectious Diseases, Emory University School of Medicine, Atlanta, GA USA; 10 Departments of Global Health, Medicine and Epidemiology, University of Washington, Seattle, WA USA; 11 Division of HIV, Infectious Diseases & Global Medicine, Zuckerberg San Francisco General, University of California, San Francisco, San Francisco, CA USA

**Keywords:** Antimicrobial resistance, Epidemiology, Translational research

## Abstract

Doxycycline post-exposure prophylaxis (doxy-PEP) reduces bacterial sexually transmitted infections among men who have sex with men and transgender women. Although poised for widespread clinical implementation, the impact of doxy-PEP on antimicrobial resistance remains a primary concern as its effects on the gut microbiome and resistome, or the antimicrobial resistance genes (ARGs) present in the gut microbiome, are unknown. To investigate these effects, we studied participants from the DoxyPEP trial, a randomized clinical trial comparing doxy-PEP use, a one-time doxycycline 200-mg dose taken after condomless sex (DP arm, *n* = 100), to standard of care (SOC arm, *n* = 50) among men who have sex with men and transgender women. From self-collected rectal swabs at enrollment (day-0) and after 6 months (month-6), we performed metagenomic DNA sequencing (DNA-seq) or metatranscriptomic RNA sequencing (RNA-seq). DNA-seq data were analyzable from 127 samples derived from 89 participants, and RNA-seq data were analyzable from 86 samples derived from 70 participants. We compared the bacterial microbiome and resistome between the two study arms and over time. The median number of doxycycline doses taken since enrollment by participants with DNA-seq data was zero (interquartile range (IQR): 0–7 doses) for the SOC arm and 42 (IQR: 27–64 doses) for the DP arm. Tetracycline ARGs were detected in all day-0 DNA-seq samples and in 85% of day-0 RNA-seq samples. The proportional mass of tetracycline ARGs in the resistome increased between day-0 and month-6 in DP participants from 46% to 51% in the metagenome (*P* = 2.3 × 10^−2^) and from 4% to 15% in the metatranscriptome (*P* = 4.5 × 10^−6^), but no statistically significant increases in other ARG classes were observed. Exposure to a higher number of doxycycline doses correlated with proportional enrichment of tetracycline ARGs in the metagenome (Spearman’s *ρ* = 0.23, *P* = 9.0 × 10^−3^) and metatranscriptome (Spearman’s *ρ* = 0.55, *P* = 3.7 × 10^−8^). Bacterial microbiome alpha diversity, beta diversity and total bacterial mass did not differ between day-0 and month-6 samples from DP participants when assessed by either DNA-seq or RNA-seq. In an abundance-based correlation analysis, we observed an increase over time in the strength of the correlation between tetracycline ARGs and specific bacterial taxa, including some common human pathogens. In sum, doxy-PEP use over a 6-month period was associated with an increase in the proportion of tetracycline ARGs comprising the gut resistome and an increase in the expression of tetracycline ARGs. At 6 months of doxy-PEP use, no residual differences were observed in alpha and beta diversity or taxonomic composition of the gut microbiome. As doxy-PEP is implemented as a public health strategy, further studies and population-level surveillance of doxycycline-resistant pathogens are needed to understand the implications of these findings. ClinicalTrials.gov registration number: NCT03980223.

## Abstract (web summary)

In participants from a randomized controlled trial, doxy-PEP use over 6 months minimally affected the gut microbiome’s taxonomic composition but increased the abundance and active expression of tetracycline antibiotic resistance genes.

## Main

Doxycycline post-exposure prophylaxis (doxy-PEP) is highly efficacious in preventing bacterial sexually transmitted infections (STIs) in randomized, controlled clinical trials among men who have sex with men (MSM) and transgender women living with HIV or on pre-exposure prophylaxis (PrEP) to prevent HIV infection^1–3^. This new public health strategy has been incorporated into guidelines for STI prevention for MSM and transgender women by the US Centers for Disease Control and Prevention (CDC)^4^, with World Health Organization guidelines^5^ in process. Widespread implementation of doxy-PEP among all MSM and transgender women could increase doxycycline consumption substantially, with high-end estimates of as much as 3.36 million doses per month in the United States^6^. As such, a primary outstanding concern is the potential for doxy-PEP to select for antimicrobial-resistant bacteria and adversely impact the human microbiome^7–9^.

Antimicrobial resistance is a major global public health challenge that complicates the management of infectious diseases^10^. The overuse and misuse of antibiotics in both human healthcare and agriculture are major contributors to this problem^11–13^. Multidrug-resistant *Neisseria gonorrhoeae*, which can be resistant to all first line antimicrobial treatments, has been increasing in prevalence and has been labeled as an urgent threat by the US CDC^14–18^. Given this, there are concerns that doxy-PEP implementation may lead to increased tetracycline-resistant *N. gonorrhoeae*, which may be less susceptible to doxy-PEP; to co-selection for beta-lactam resistance in *N. gonorrhoeae*, which is currently the first-line antibiotic treatment; and to selection for antimicrobial resistance in commensal organisms as well as disease-causing pathogens, such as *Staphylococcus aureus*.

The few studies that have evaluated the impact of doxycycline exposure on the human microbiome involved daily doxycycline use and were based primarily on bacterial culture or 16S rRNA gene amplicon sequencing, limiting their ability to evaluate the antimicrobial resistance genes (ARGs) in the microbiome, termed the resistome^19–22^. In contrast, metagenomic DNA sequencing (DNA-seq) allows for comprehensive assessment of bacterial genomes and the genetic potential for antimicrobial resistance, and metatranscriptomic RNA sequencing (RNA-seq) provides a functional profile of actively transcribed genes, including ARGs.

To address the outstanding question of whether doxy-PEP impacts the ecology of the gut microbiome and resistome, we studied longitudinally collected rectal swabs from DoxyPEP clinical trial participants using a combination of DNA-seq and RNA-seq approaches. We found that doxy-PEP use increased the proportion and expression of tetracycline resistance genes in the gut microbiome while minimally affecting community composition and diversity. Together, our findings provide new insight into the microbiological impacts of doxy-PEP before its widespread deployment for STI prevention.

## Results

### Clinical cohort

**Fig. 1:**
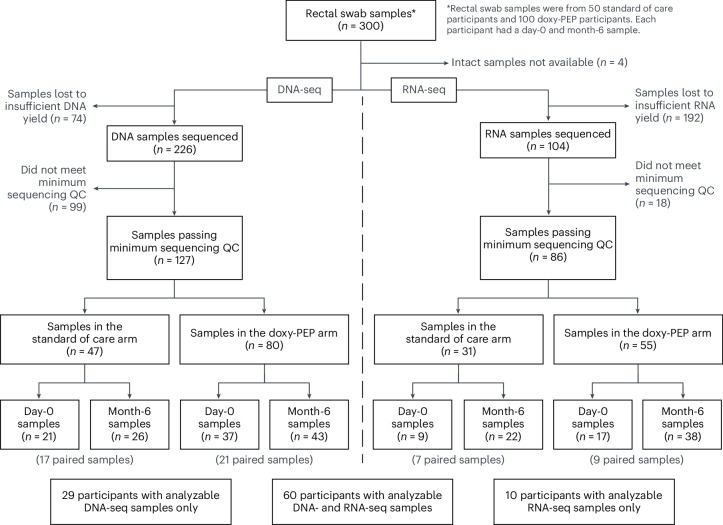
Flow diagram of the SOC and DP participant samples received, sequenced and used for analysis. QC, quality control.

We studied 100 doxy-PEP (DP) and 50 standard of care (SOC) participants from the 501 participants enrolled in the DoxyPEP clinical trial^2^, and we performed DNA-seq and RNA-seq on rectal swabs self-collected at time of enrollment and after 6 months. We preferentially evaluated participants with the highest reported doxy-PEP use. Among the selected participants, 89 had analyzable DNA-seq samples (58 day-0 samples, 69 month-6 samples), and 70 had analyzable RNA-seq samples (26 day-0 samples, 60 month-6 samples) (Fig. 1). No significant differences were observed between participants in the DP arm versus the SOC arm with regard to age, race/ethnicity, education level, housing situation or proportion of participants living with HIV (Table 1). Among the 69 participants with month-6 DNA-seq data, the median number of doxycycline doses taken since enrollment was zero (interquartile range (IQR): 0–7 doses) for the SOC arm and 42 (IQR: 27–64 doses) for the DP arm (Table 1); the median number of doxycycline doses taken per month by the DP participants in the DoxyPEP clinical trial was four doses, or 24 doses over 6 months^2^. For the 60 participants with month-6 RNA-seq samples, the median number of doxycycline doses in the SOC arm was also zero (IQR: 0–7 doses) as compared to 42 (IQR: 29–65 doses) in the DP arm (Table 1). Participants in the SOC arm were twice as likely to have received a cephalosporin during the 6-month period, and some SOC participants received doxycycline for clinical indications, such as STI treatment.

**Table 1:** Participant characteristics

A								
Participant characteristics	DNA-seq				RNA-seq			
	SOC *n* = 30	DP *n* = 59	Total *n* = 89	*P* value	SOC *n* = 24	DP *n* = 46	Total *n* = 70	*P* value
Age (years): median (IQR)	39 (31–49)	38 (32–50)	38 (32–50)	0.86	39 (31–50)	40 (31–54)	39 (31–53)	0.58
Race/ethnicity				0.87				0.89
Non-Hispanic White	14 (47%)	30 (51%)	44 (49%)		10 (42%)	24 (52%)	34 (49%)	
Hispanic White	4 (13%)	11 (19%)	15 (17%)		4 (17%)	9 (20%)	13 (19%)	
Asian/Pacific Islander	3 (10%)	7 (12%)	10 (11%)		2 (8%)	3 (7%)	5 (7%)	
Black/African American	2 (7%)	2 (3%)	4 (4%)		2 (8%)	2 (4%)	4 (6%)	
Multiracial/Other	6 (20%)	7 (12%)	13 (15%)		5 (21%)	6 (13%)	11 (16%)	
Unknown	1 (3%)	2 (3%)	3 (3%)		1 (4%)	2 (4%)	3 (4%)	
Living with HIV infection	12 (40%)	23 (39%)	35 (39%)	0.93	9 (38%)	24 (52%)	33 (47%)	0.24
Unknown CD4 count	4/12 (33%)	4/23 (17%)	8/35 (23%)	0.40	2/9 (22%)	5/24 (21%)	7/33 (21%)	1.00
CD4 count (cells per mm^3^): median (IQR)	710 (398–790)	646 (524–807)	695 (495–807)	0.83	743 (508–984)	726 (577–850)	734 (562–896)	0.79

B						
Month-6 participant characteristics	DNA-seq (*n* = 69 participants)			RNA-seq (*n* = 60 participants)		
	SOC *n* = 26	DP *n* = 43	*P* value	SOC *n* = 22	DP *n* = 38	*P* value
Doxycycline doses: median (IQR, range)	0 (0–7, 0–14)	42 (27–64, 0–200)	4.2 × 10^−10^	0 (0–7, 0–14)	42 (29–65, 0–200)	1.9 × 10^−9^
0 doses	16 (62%)	3 (7%)	3.0 × 10^−10^	14 (64%)	2 (5%)	2.3 × 10^−9^
1–25 doses	10 (38%)	8 (19%)		8 (36%)	7 (18%)	
26–50 doses	0 (0%)	15 (35%)		0 (0%)	15 (39%)	
>50 doses	0 (0%)	17 (40%)		0 (0%)	14 (37%)	
Received non-doxycycline antibiotics since enrollment	15 (58%)	14 (33%)	5.2 × 10^−7^	13 (59%)	10 (26%)	7.1 × 10^−10^
Cephalosporin	12 (46%)	10 (23%)	4.8 × 10^−2^	10 (45%)	8 (21%)	4.7 × 10^−2^
Penicillin	3 (12%)	3 (7%)	0.67	3 (14%)	2 (5%)	0.35
Clindamycin	0 (0%)	2 (5%)	0.52	0 (0%)	1 (3%)	1.00
Azithromycin	0 (0%)	1 (2%)	1.00	1 (5%)	0 (0%)	0.37
Quinolone	0 (0%)	1 (2%)	1.00	0 (0%)	1 (3%)	1.00
Vancomycin	0 (0%)	1 (2%)	1.00	0 (0%)	1 (3%)	1.00

**A**, Participant characteristics at time of enrollment by analyzable DNA-seq and RNA-seq samples. **B**, Participant characteristics for those with month-6 samples by DNA-seq and RNA-seq. Each doxycycline treatment day was equivalent to one doxycycline dose. Doxy-PEP dose was a one-time doxycycline dose of 200 mg. *P* values were calculated by Wilcoxon rank-sum test for age, CD4 count and number of doxycycline doses and by the chi-square test or Fisher’s exact test if count was less than 5 for all other variables.

### Impact of doxy-PEP on the gut antimicrobial resistome

**Extended Data Fig. 1:**
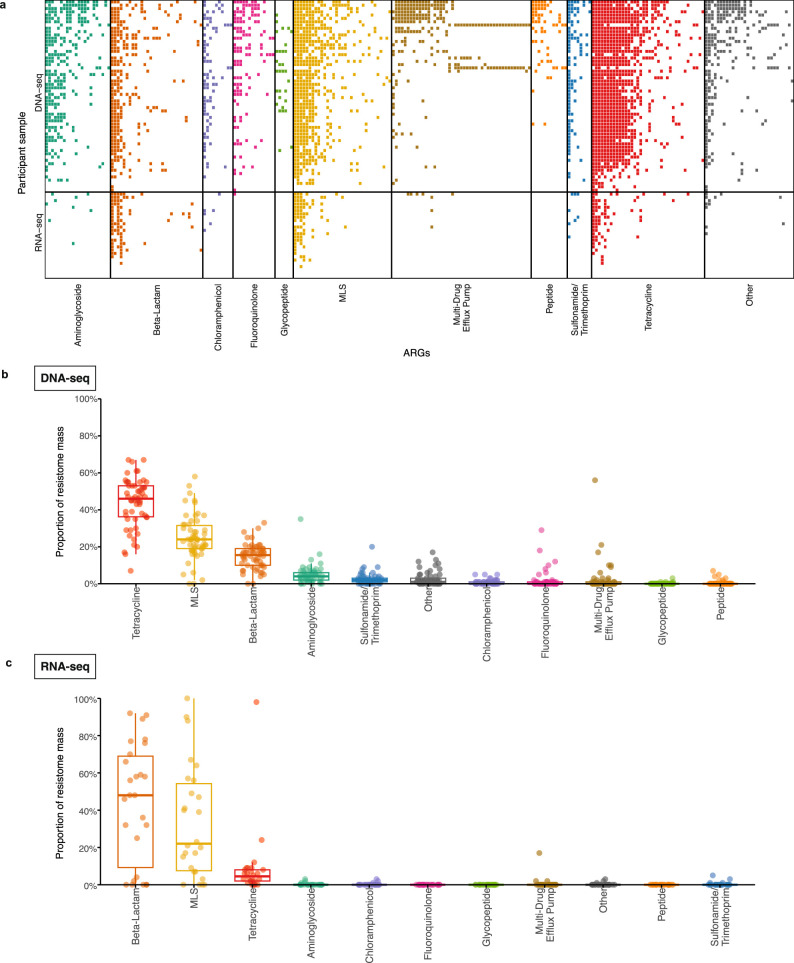
Resistome characteristics at enrollment. **a**) Heatmap of the antimicrobial resistance genes (ARGs) detected in DNA-seq samples (n = 58) and RNA-seq samples (n = 26) at enrollment by ARG class. Proportion of the resistome mass by ARG class on enrollment in (**b**) DNA-seq samples (n = 58) and (**c**) RNA-seq samples (n = 26). Boxplot elements include a center line (median), box limits (upper and lower quartiles), whiskers (1.5x interquartile range). Abbreviation: ARG, antimicrobial resistance gene; MLS, macrolide-lincosamide-streptogramin.

We first assessed the presence of tetracycline resistance genes in the resistome at enrollment. Among day-0 samples, a total of 41 tetracycline resistance genes were detected by DNA-seq and 17 tetracycline resistance genes by RNA-seq. Tetracycline ARGs were the most prevalent ARG class in the resistome, with at least one tetracycline ARG detected in 100% (*n* = 58/58) of samples by DNA-seq and in 85% (*n* = 22/26) of samples by RNA-seq (Extended Data Fig. 1a). Tetracycline ARGs represented the largest proportion (46%) of the resistome by mass (Extended Data Fig. 1b) but accounted for only 4% of the expressed resistome mass at the time of enrollment (Extended Data Fig. 1c).

**Fig. 2:**
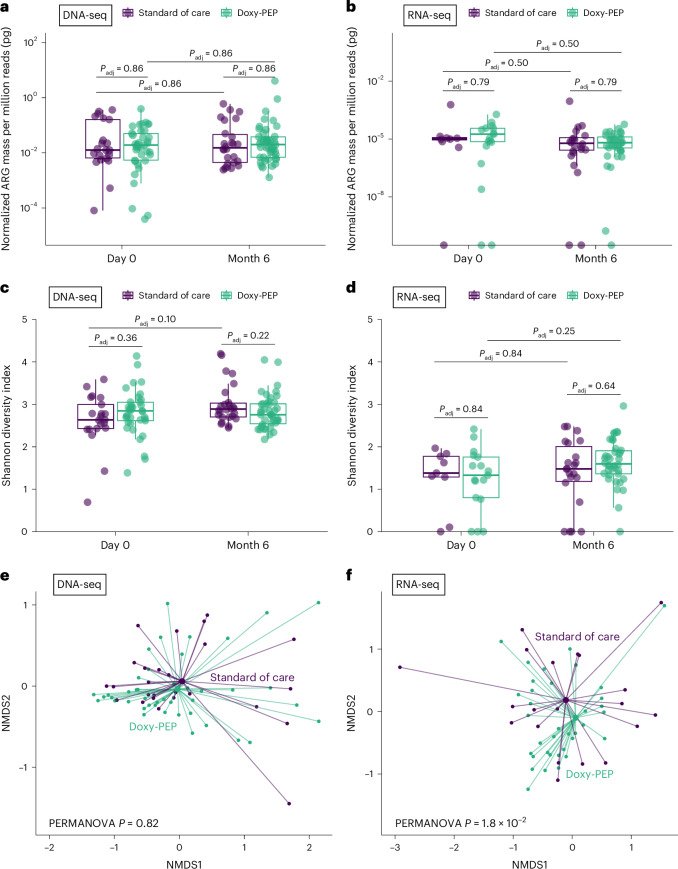
Impact of doxy-PEP use on the gut resistome for DNA-seq samples and RNA-seq samples. **a**–**d**, Normalized resistome mass (**a**,**b**) and resistome alpha diversity (Shannon diversity index) (**c**,**d**) for DP versus SOC participants (DNA-seq: *n* = 127; RNA-seq: *n* = 86). **e**,**f**, Resistome beta diversity (Bray–Curtis index) in DP versus SOC participants at 6 months (DNA-seq: *n* = 69; RNA-seq: *n* = 60). *P* values were calculated using the two-sided Wilcoxon rank-sum test and adjusted for multiple comparisons (**a**–**d**). Two-sided PERMANOVA *P* values for beta diversity were calculated and adjusted for multiple comparisons (**e**,**f**). Box plot elements include a center line (median), box limits (upper and lower quartiles) and whiskers (1.5× IQR).

We evaluated for ecological differences in the resistome between the DP and SOC arms by assessing resistome: (1) mass, computed from spiked-in mass standards; (2) alpha diversity, measured by Shannon diversity index; and (3) beta diversity, measured by Bray–Curtis dissimilarity index. No differences between DP and SOC arms were observed in resistome mass (Fig. 2a,b) or alpha diversity (Fig. 2c,d) at any timepoints by either DNA-seq or RNA-seq. Although no differences in beta diversity were observed by DNA-seq (Fig. 2e), significant compositional differences in the expressed resistome were found between the DP and SOC arms by RNA-seq at month 6 (adjusted *P* (*P*_adj_) = 1.8 × 10^−2^ by PERMANOVA; Fig. 2f).

**Fig. 3:**
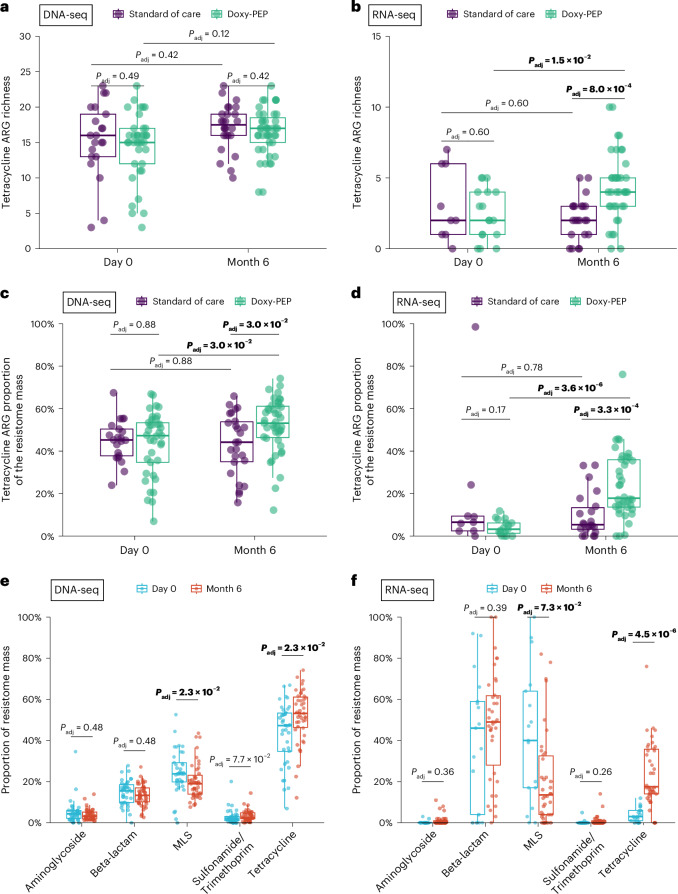
Impact of doxy-PEP use on tetracycline and non-tetracycline ARGs for DNA-seq samples and RNA-seq samples. Tetracycline ARG richness (**a**,**b**) and tetracycline ARG proportion of resistome mass (**c**,**d**) were compared between SOC and DP arms at each visit and over time (DNA-seq: *n* = 127; RNA-seq: *n* = 86). **e**,**f**, The proportion of the resistome mass by ARG classes over time within the DP arm (DNA-seq: *n* = 80; RNA-seq: *n* = 55). *P* values were calculated using the two-sided Wilcoxon rank-sum test and adjusted for multiple comparisons. Box plot elements include a center line (median), box limits (upper and lower quartiles) and whiskers (1.5× IQR).

**Extended Data Fig. 2:**
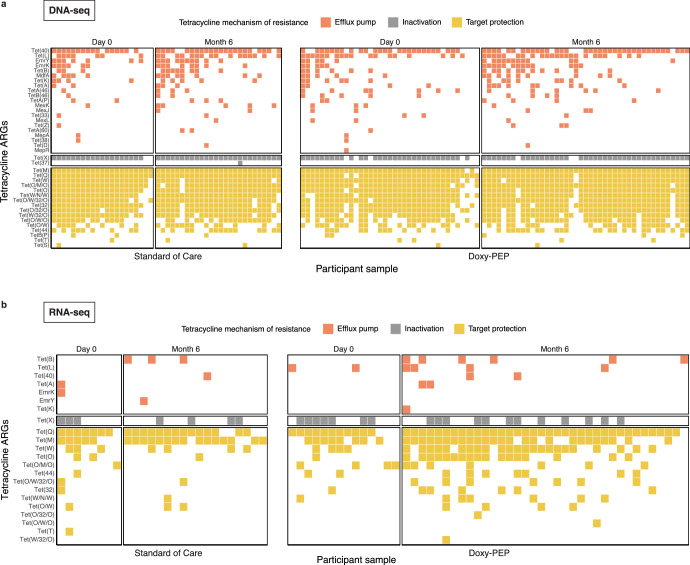
Heatmaps of tetracycline antimicrobial resistance genes (ARGs) detected by mechanism of action, visit (day-0 or month-6), and study arm (standard of care, SOC, or doxy-PEP) by (a) DNA-seq or (b) RNA-seq. DNA-seq: n = 127 samples; RNA-seq: n = 86 samples.

We further evaluated the impact of doxy-PEP use on different ARG classes by comparing the day-0 and month-6 samples within the DP arm. Although tetracycline ARG richness was not found to differ over time by DNA-seq (*P*_adj_ = 0.12; Fig. 3a), the number of detectably expressed tetracycline ARGs increased by RNA-seq (*P*_adj_ = 1.5 × 10^−2^; Fig. 3b). Among participants in the DP arm, the proportion of tetracycline ARGs in the resistome identified by DNA-seq increased over the 6-month study period (46% to 51%, *P*_adj_ = 3.0 × 10^−2^; Fig. 3c) as did the proportion of expressed tetracycline ARGs identified by RNA-seq (4% to 15%, *P*_adj_ = 3.6 × 10^−6^; Fig. 3d). The most common mechanism of tetracycline resistance observed was ribosomal target protection in both the metagenome and metatranscriptome (Extended Data Fig 2a,b).

**Extended Data Fig. 3:**
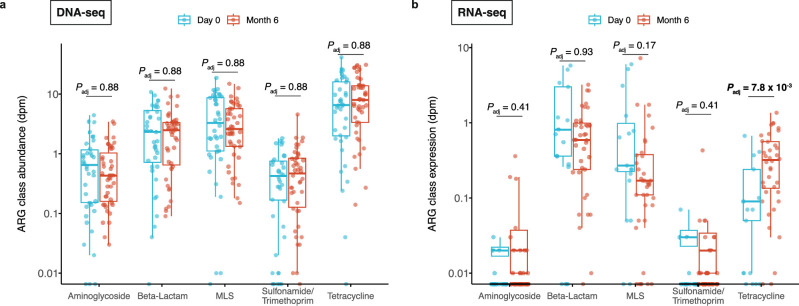
Impact of Doxy-PEP use on abundance of tetracycline and non-tetracycline antimicrobial resistance genes (ARGs) for DNA-seq samples and RNA-seq samples. ARG class abundance over time within the doxy-PEP arm for (**a**) DNA-seq samples (n = 80) and (**b**) RNA-seq samples (n = 55). P-values were calculated using the two-sided Wilcoxon rank-sum test and adjusted for multiple comparisons. Boxplot elements include a center line (median), box limits (upper and lower quartiles), whiskers (1.5x interquartile range). Significant p-values (<0.05) are bolded. Abbreviations: Padj, adjusted p-value.

No proportional increases were noted in other non-tetracycline ARG classes (Fig. 3e,f), suggesting specificity of doxy-PEP use for tetracycline ARGs. Although no change in tetracycline ARG abundance was observed by DNA-seq over time, tetracycline ARG expression by RNA-seq significantly increased in DP participants over 6 months of follow-up (Extended Data Fig. 3). A sensitivity analysis adjusting for HIV status and cephalosporin exposure days demonstrated statistically significant increases in proportional mass of tetracycline ARGs and decreases in proportional mass of macrolide-lincosamide-streptogramin (MLS) ARGs between day 0 and month 6 in the DP arm for both DNA-seq and RNA-seq data (Supplementary Table 1). We also evaluated for specific beta-lactam resistance genes of high public health concern at enrollment and at month 6. The extended-spectrum beta-lactamase-encoding gene *CTX-M* was detected at month 6 in one SOC participant by both DNA-seq and RNA-seq and in three DP participants (one by DNA-seq and RNA-seq, one by DNA-seq only and one by RNA-seq only); *CTX-M* was not detected in any day-0 samples by either DNA-seq or RNA-seq. The carbapenemase genes *KPC*, *NDM*, *VIM* and *OXA-48* were not detected in any samples by DNA-seq or RNA-seq.

**Fig. 4:**
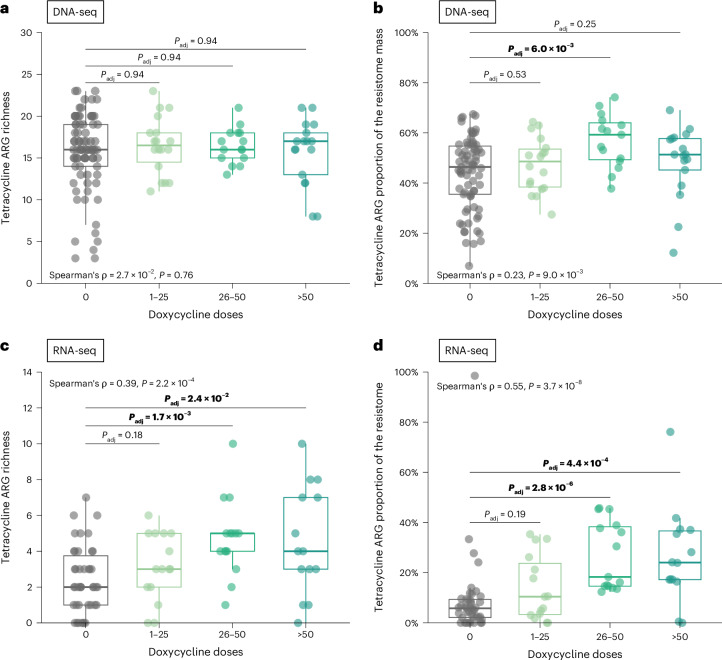
Impact of doxy-PEP use on tetracycline ARGs by number of doxycycline doses received for DNA-seq samples and RNA-seq samples. A test of trend was used to compare tetracycline ARG richness (**a**,**c**) and proportion of tetracycline ARG mass to resistome mass by number of doxycycline doses received (**b**,**d**) (DNA-seq: *n* = 127; RNA-seq: *n* = 86). *P* values were calculated using the two-sided Wilcoxon rank-sum test and adjusted for multiple comparisons. The two-sided Spearman’s rank correlation test was used to calculate Spearman’s ρ and *P* value. Box plot elements include a center line (median), box limits (upper and lower quartiles) and whiskers (1.5× IQR).

**Extended Data Fig. 4:**
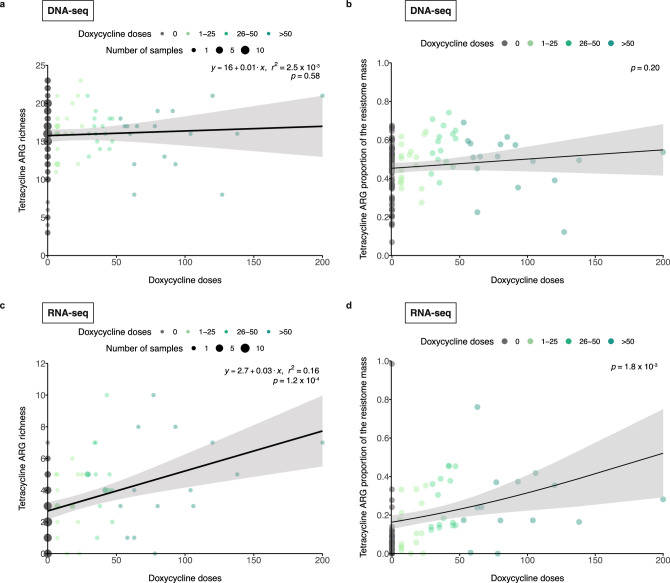
Impact of Doxy-PEP use on tetracycline ARGs by number of doxycycline doses received for DNA-seq samples (a, b) and RNA-seq samples (c, d). (**a, c**) Tetracycline ARG richness and (**b, d**) proportion of tetracycline ARG mass within the resistome by number of doxycycline doses received was plotted and fitted to a linear regression line for the outcome of tetracycline ARG richness and an inflated beta-regression line for the outcome of proportional tetracycline mass (DNA-seq: n = 127 samples, RNA-seq: n = 86 samples). The regression model is shown as a line, and the gray shaded areas surrounding the regression lines show the 95% confidence intervals. P-values from the regression models are obtained by two-sided Wald tests. Significant p-values (<0.05) are bolded. Significant p-values (<0.05) are bolded. Abbreviations: ARG, antimicrobial resistance gene; p.adj, adjusted p-value.

We next asked whether doxycycline influenced tetracycline ARGs in a dose-dependent manner. In the metagenome, the number of doxycycline doses was not associated with changes in richness of tetracycline ARGs (Spearman’s *ρ* = 2.7 × 10^−3^, *P* = 0.76; Fig. 4a). However, it was weakly positively correlated with the proportion of tetracycline ARGs in the resistome (Spearman’s *ρ* = 0.23, *P* = 9.0 × 10^−3^; Fig. 4b), potentially indicating preferential growth of tetracycline ARG-carrying bacteria. Furthermore, in the metatranscriptome, the number of doxycycline doses was strongly positively correlated with both tetracycline ARG richness (Spearman’s *ρ* = 0.39, *P* = 2.2 × 10^−4^) and the relative proportion of expressed tetracycline ARGs in the resistome (Spearman’s *ρ* = 0.55, *P* = 3.7 × 10^−8^; Fig. 4c,d). We noted that only participants who had reported taking more than 25 doxycycline doses over the 6-month follow-up period demonstrated significantly increased tetracycline ARG richness and proportional tetracycline ARG representation compared to those who had not taken any doxycycline (Fig. 4c,d). Sensitivity linear regression models with the number of doxycycline doses demonstrated similar results, with the exception that doxycycline dose exposure as a continuous variable was no longer found to be significantly correlated with proportional tetracycline ARG mass in the metagenome (Extended Data Fig. 4). In the metatranscriptome, the regression models continued to demonstrate a significant positive correlation between doxycycline dose exposure when compared to tetracycline ARG richness (*P* = 1.2 × 10^−4^) and proportional tetracycline ARG mass (*P* = 1.8 × 10^−3^) (Extended Data Fig. 4).

**Fig. 5:**
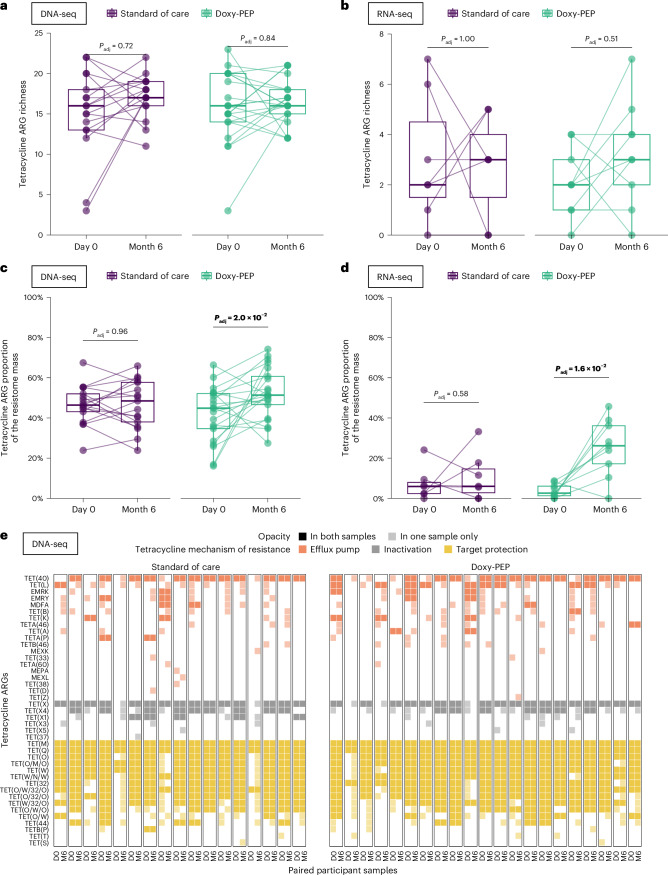
Impact of doxy-PEP use on tetracycline ARGs in sets of paired DNA-seq samples and paired RNA-seq samples. Tetracycline ARG richness (**a**,**b**) and the tetracycline ARG proportional mass within the resistome (**c**,**d**) were compared between SOC and DP arms by visit and over time (DNA-seq: *n* = 38 paired sample sets; RNA-seq: *n* = 16 paired sample sets). **e**, Heatmap of tetracycline ARGs detected by DNA-seq in paired samples (day-0 and month-6 samples) for the SOC and DP arms (*n* = 38 paired sample sets). *P* values were calculated using the two-sided Wilcoxon signed-rank test for paired samples and adjusted for multiple comparisons. Box plot elements include a center line (median), box limits (upper and lower quartiles) and whiskers (1.5× IQR). D0, day 0; M6, month 6.

We performed a secondary analysis with paired samples (DNA-seq: 38 participants; RNA-seq: 16 participants) (Fig. 1). Among the paired samples, no changes in tetracycline ARG richness were noted (Fig. 5a,b). In the DP arm, however, we observed a significant increase in the proportion of tetracycline ARGs in the resistome when measured by either DNA-seq (45% to 51%, *P*_adj_ = 2.0 × 10^−2^) or RNA-seq (6% to 26%, *P*_adj_ = 1.6 × 10^−2^) (Fig. 5c,d). We noted that, in both the SOC and DP arms, tetracycline ARGs with diverse mechanisms of action were both lost and gained between the day-0 and month-6 paired samples (Fig. 5e).

### Impact of doxy-PEP on the gut microbiome and transcriptome

**Extended Data Fig. 5:**
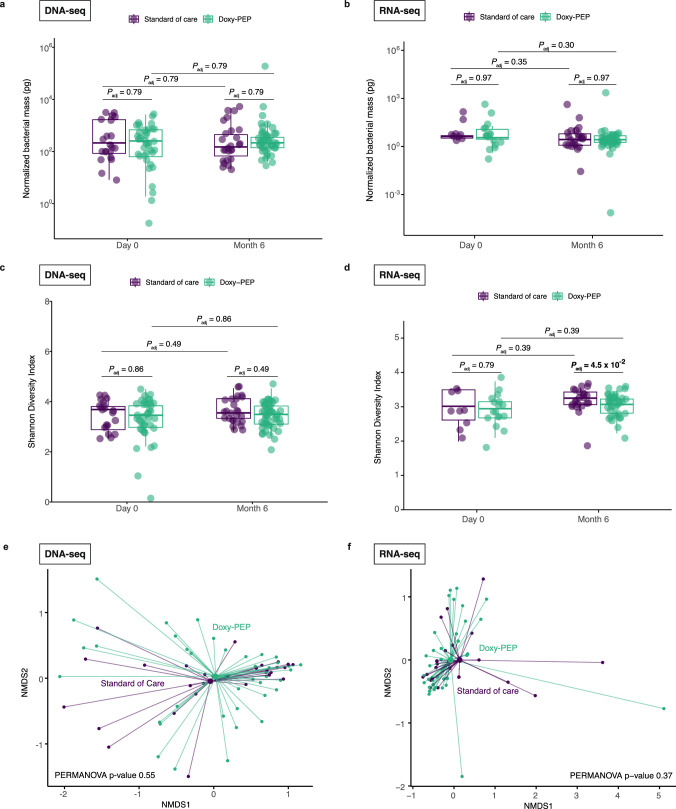
Bacterial taxonomic metrics for DNA-seq samples (a, c, e) and RNA-seq samples (b, d, f). (**a, b**) Normalized bacterial microbiome mass and (**c, d**) alpha diversity (Shannon Diversity Index) for doxy-PEP versus standard of care (SOC) participants (DNA-seq: n = 127 samples, RNA-seq: n = 86 samples). (**e, f**) Bacterial microbiome beta diversity (Bray-Curtis Index) in doxy-PEP versus SOC participants at six months (DNA-seq: n = 69 samples, RNA-seq: n = 60 samples). For figures a-d, p-values for bacterial microbiome mass and alpha diversity were calculated using the two-sided Wilcoxon rank-sum test and adjusted for multiple comparisons; boxplot elements include a center line (median), box limits (upper and lower quartiles), whiskers (1.5x interquartile range). For figures e and f, p-values for beta diversity were calculated using the two-sided PERMANOVA test and adjusted for multiple comparisons. Significant p-values (<0.05) are bolded. Abbreviations: Padj, adjusted p-value; NMDS, non-metric multidimensional scaling.

Having observed an impact of doxy-PEP on the resistome, we next evaluated the effects on gut microbial communities. We found no differences in normalized bacterial mass of the gut microbiome (Extended Data Fig. 5a) or metatranscriptome (Extended Data Fig. 5b) between the DP and SOC arms at day 0 or month 6 or within study arms between timepoints. In addition, no differences were observed in bacterial taxonomic alpha diversity between arms or timepoints (Extended Data Fig. 5b). In the metatranscriptome, although we observed increased alpha diversity at month 6 in the SOC arm compared to the DP arm (*P*_adj_ = 4.5 × 10^−2^), no differences in alpha diversity were observed at enrollment between arms or over time (Extended Data Fig. 5c). Finally, we tested for differences in microbial community composition but found no differences in beta diversity between the SOC and DP arms at the month-6 timepoint by DNA-seq or RNA-seq (Extended Data Fig. 5d,e).

**Extended Data Fig. 6:**
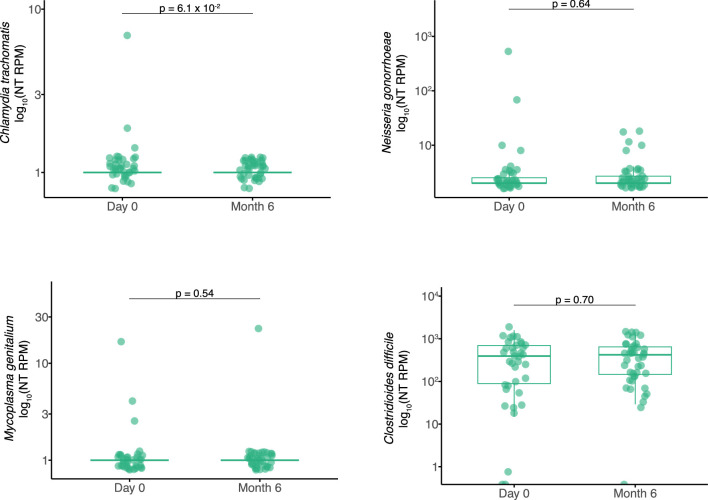
Differential abundance comparison of specific pathogens of interest between time points in the in DNA-seq samples of the doxy-PEP arm (n = 80). Bacterial abundance of several pathogens of interest (*Chlamydia trachomatis*, *Neisseria gonorrhoeae*, *Mycoplasma genitalium*, *and Clostroidioides difficile*) were compared between day-0 and month-6 samples in the doxy-PEP arm. P-values were calculated using the Wilcoxon rank-sum test and adjusted for multiple comparisons. Boxplot elements include a center line (median), box limits (upper and lower quartiles), whiskers (1.5x interquartile range). Abbreviation: NT RPM, nucleotide reads per million.

We next carried out a differential abundance analysis of bacterial taxa between enrollment and the month-6 timepoint in the DP arm using DNA-seq data. Using a consensus approach of three different differential abundance analysis methods, no bacterial genera were consistently differentially abundant between the two timepoints. We confirmed that no differences in the relative abundance of the well-known enteric and STI pathogens *Clostridium* *difficile, N. gonorrheae* or *Mycoplasma genitalium* existed between enrollment and month 6 in DP participants (Extended Data Fig. 6). We did, however, observe a possible reduction in *Chlamydia trachomatis* abundance (*P* = 0.06; Extended Data Fig. 6).

### Correlations within the resistome and microbiome

**Extended Data Fig. 7:**
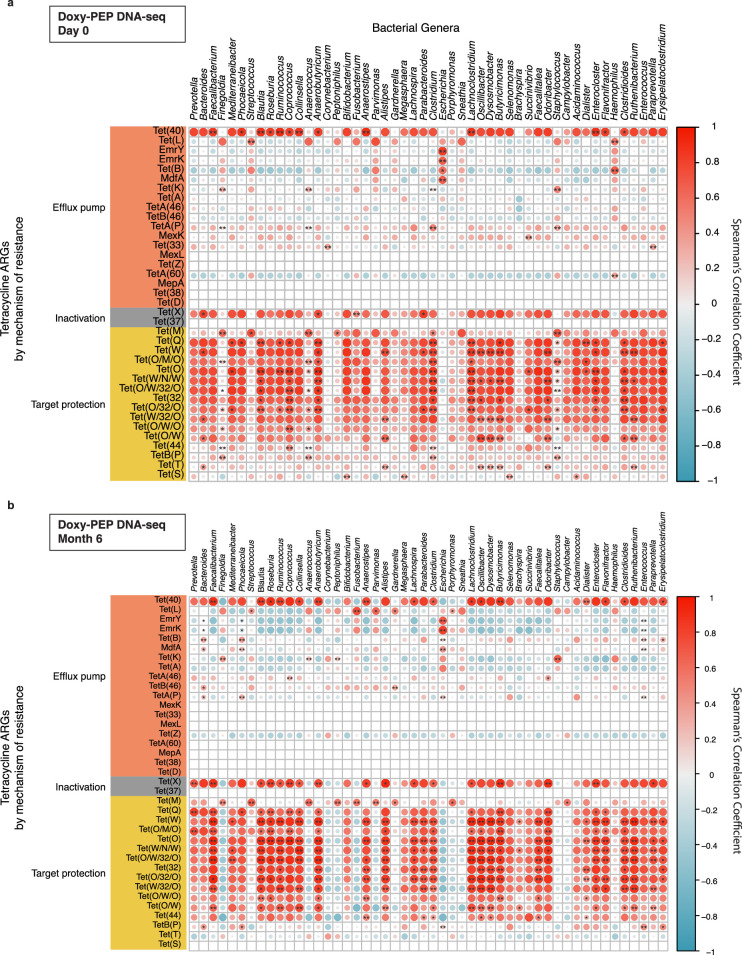
Correlation plots between the tetracycline antimicrobial resistance genes (ARGs) detected in the bacterial microbiome and the 50 most abundant bacterial genera detected in the doxy-PEP DNA-seq samples at (a) day-0 and (b) month-6. Day-0: n = 58; month-6: n = 69. Color and size of the points represent the strength of correlation between the tetracycline ARG abundance and the bacterial genera abundance as calculated with the two-sided Spearman’s correlation test. Correlations with significant p-values, adjusted for multiple comparisons, are denoted with asterisks (p < 0.05*, p < 0.01**). Tetracycline ARGs with an empty row represented cases where there was not enough data to evaluate for correlation.

**Extended Data Fig. 8:**
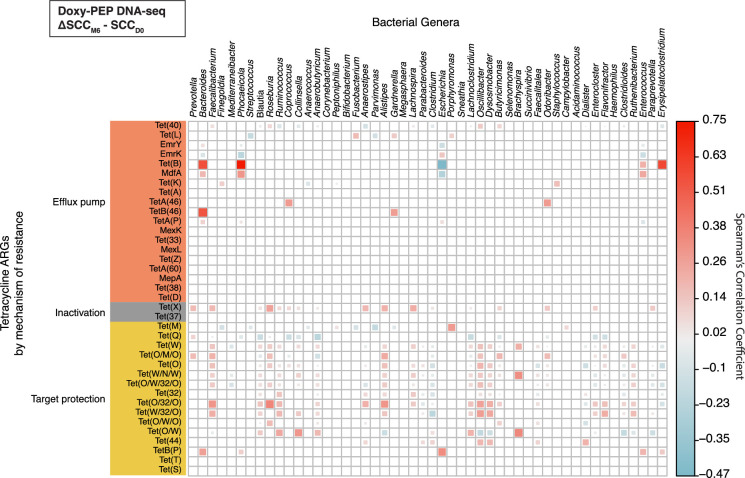
The change in Spearman’s correlation coefficient (SCC) from day-0 to month-6 between the tetracycline antimicrobial resistance genes (ARGs) detected in the bacterial microbiome and the 50 most abundant bacterial genera detected in the doxy-PEP DNA-seq samples. Day-0: n = 58; month-6: n = 69. Color and size of the square filling represent the degree of change in the correlation coefficient. Blank squares represent cases where there was not a significant correlation between the tetracycline ARG and the bacterial taxa at month 6, or there was not enough data to evaluate for correlation.

To identify linkages between the abundances of tetracycline ARGs and bacterial taxa within the gut microbiome, we performed multi-dimensional correlation analysis of paired day-0 and month-6 DNA-seq samples from DP participants (Extended Data Fig. 7a,b). Significant positive correlations were found between several tetracycline ARGs and bacterial genera, both pathogenic and commensal, at day 0 and month 6; no statistically significant negative correlations were noted. To understand how the strength of the correlations between tetracycline ARGs and bacterial taxa changed over time in the setting of doxy-PEP use, we plotted the change in Spearman’s correlation coefficient (SCC) between day 0 and month 6 (Extended Data Fig. 8). Many well-established commensal genera (for example, *Faecalibacterium* and *Gardnerella*) exhibited increased correlations with tetracycline target protection ARGs over time. In addition, some pathogenic bacteria genera (for example, *Bacteroides* and *Escherichia*) demonstrated a moderate to strong positive increase in correlation strength to individual tetracycline ARGs from the day-0 to month-6 samples. *Staphylococcus* spp. were associated with a small increase in correlation strength to Tet(K), which encodes a tetracycline efflux pump, between the day-0 and the month-6 samples.

## Discussion

In patients from a randomized controlled trial^2^, doxy-PEP use over 6 months minimally affected the taxonomic composition of the gut bacterial microbiome. However, we found a significant expansion of tetracycline ARGs in the resistome and a dose-dependent increase in their active expression. Notably, the impact of doxy-PEP was restricted to tetracycline class ARGs, without evidence of co-selection for genes conferring resistance to other antibiotic classes. The clinical implications of the tetracycline ARG expansion in the gut resistome require further investigation.

A healthy gut microbiota is essential for host metabolism, immunity and intestinal barrier function^23^. Disruptions in the gut microbiome can lead to growth of pathogenic or resistant organisms^24^, increased susceptibility to infection^25^ and increased risk of non-communicable diseases, such as obesity and cardiovascular disease^26^. In this cohort, we found that doxy-PEP use over 6 months did not significantly alter gut microbiome alpha diversity, beta diversity or mass. Despite stability of these community-level measures, differential abundance analyses demonstrated no taxonomic shifts over the 6 months of doxy-PEP use. Our findings are consistent with two prior culture-based studies of long-term daily low-dose (20 mg twice daily) doxycycline use, which demonstrated minimal changes in gut microbiota^21,22^. A recent metagenomic study evaluating the impact of long-term daily doxycycline exposure, however, found significant alterations in the composition of skin microbiota, with more varying effects on the oral and fecal communities^27^. Although we did not find substantial compositional gut microbiome shifts, it is possible that measurable changes may have been observed if we were able to account for time between doxy-PEP exposure and the sample collection in the analyses. Additionally, other anatomical sites, such as the skin or the respiratory tract, may have experienced more significant perturbations in the microbiomes.

Tetracycline ARGs were the most prevalent and abundant ARG class represented in the gut microbiome, comprising 46% of ARG mass even before doxy-PEP exposure, a finding consistent with observations from worldwide population studies of the human gut microbiome^28,29^. The widespread prophylactic use of tetracyclines in livestock selects for tetracycline-resistant organisms and may contribute to the predominance of tetracycline ARGs in the human gut microbiome^30,31^, along with tetracycline use for treatment of STIs and other indications, which is expected to be common in this study population. Over the last 50 years, tetracycline resistance among *Bacteroides* species has increased from 30% to more than 80%, hypothesized to be driven by horizontal transfer of tetracycline ARGs within the gut microbiome^32^.

We found that intermittent doxy-PEP use led to a small (46% to 51%) but significant proportional expansion of tetracycline ARGs in the gut resistome. These findings are consistent with several studies demonstrating increases in both tetracycline-resistant bacteria^21,22,27^ and tetracycline ARG abundance^19^ after daily doxycycline use. Potential explanations for this finding include selective pressure from doxycycline exposure driving either the gain of new tetracycline ARGs or the elimination of susceptible bacteria and expansion of tetracycline-resistant bacteria. Because we did not find that doxy-PEP use led to a significant increase in tetracycline ARG richness over the 6-month study period in the DNA-seq data, the observed increase in tetracycline ARG proportional representation likely reflects expansion of previously existing resistance genes and their associated bacteria rather than the acquisition of new tetracycline ARGs.

Because multiple ARGs can be found together on the same plasmid, antibiotic exposure in some cases can co-select for resistance to multiple drug classes. The data from this cohort suggest that co-selection for resistance to multiple antibiotic classes did not readily occur in the setting of doxy-PEP. Although we found that doxy-PEP use led to a significant increase in both the active transcription of tetracycline ARGs and their proportional expansion in the resistome, other classes of ARGs, including specific ARGs of public health concern, were largely unaffected.

The impacts of doxy-PEP use were much more striking at the transcriptional level and highlight the additional benefits of assessing the microbiome using both DNA-seq and RNA-seq. Specifically, we found a dose-dependent increase in both tetracycline ARG expression and proportional representation of tetracycline ARGs in the metatranscriptome. In the DoxyPEP clinical trial^2^, the intervention arm reported using a median of 24 doxycycline doses over 6 months. Notably, we observed significant impacts on the resistome only in participants who took 25 or more doxycycline doses over 6 months, suggesting that not all doxy-PEP users may have noticeable changes to their gut resistome after 6 months of doxy-PEP use. These findings are in line with a recent study evaluating the impact of low-dose and high-dose doxycycline regimens (20 mg twice daily versus 100 mg twice daily for 56 d) on skin flora, which found that the high-dose regimen was associated with more emergence, selective expansion and persistence of doxycycline-resistant staphylococci on the skin at the 1-year follow-up^27^. Interestingly, doxycycline-resistant *Staphylococcus epidermidis* isolates recovered from individuals receiving the low-dose regimen had lower minimum inhibitory concentrations of doxycycline compared to isolates recovered from individuals receiving the high-dose regimen, supporting the idea that dose and frequency^27,33^ of doxycycline exposure may contribute to the emergence of resistance.

The relationship between detection of tetracycline ARGs and phenotypic antimicrobial resistance is not well understood. The DoxyPEP trial^2^ found an absolute proportional increase in tetracycline-resistant *N. gonorrhoeae* infections at 12 months, although the overall number of isolates was small, and statistical comparisons were not performed. In an effort to understand which bacteria were associated with the expansion in tetracycline ARGs, we performed exploratory abundance-based correlation analyses between tetracycline ARGs and bacterial genera in the gut microbiome. We found significant and increasing correlations between tetracycline efflux pump ARGs and the abundance of several genera encompassing clinically relevant human pathogens, including *Bacteroides*, *Escherichia* and, to a smaller extent, *Staphylococcus*, in DP participants after 6 months of doxycycline use. These results suggest that at least some of the tetracycline ARG expansion may be associated with potential bacterial pathogens, and surveillance should assess whether these changes are associated with increases in clinically significant doxycycline-resistant infections.

In addition, we observed abundance-based correlations between tetracycline ARGs and enteric commensals, with correlations increasing in strength from day 0 to month 6 in the DP arm. The gut microbiome is a well-known reservoir of ARGs^34^ that facilitates horizontal gene transfer between commensal and pathogenic bacteria^35,36^. This is of particular concern as many tetracycline ARGs are associated with plasmids, transposons and other mobile genetic elements^37^, which could facilitate their transfer to pathogenic bacteria carried in the gut microbiome. Further studies using long-read sequencing or high-throughput chromosome conformation capture (Hi-C) metagenomic sequencing^38^ and experimental mouse models are needed to definitively assess connections between specific bacterial taxa and tetracycline ARGs in the setting of doxy-PEP.

Strengths of this study include leveraging a clinical trial to carry out the first, to our knowledge, in-depth assessment of doxy-PEP use on the gut microbiome and resistome; to provide detailed information on participant-reported doxycycline use enabling dose–response analyses; and to combine metagenomics and metatranscriptomics to assess both the presence and active transcription of microbes and their ARGs. Furthermore, to our knowledge, this is the largest antimicrobial resistome study to date evaluating the impacts of doxycycline—a widely used broad-spectrum antibiotic for the treatment and prophylaxis of human and animal infectious diseases.

We also acknowledge the limitations of this study. First, samples represented only a subset of the DoxyPEP clinical trial participants over the first 6 months of follow-up, and, in the DP arm, samples from participants with higher doxy-PEP use were preferentially selected, which may have biased findings away from the null compared to the average individual using doxy-PEP. Second, some participants from the SOC arm received doxycycline for STI treatment or other clinical indications, which may have biased the findings toward the null. Third, our analyses were limited by the quality of the self-collected rectal swabs; many specimens did not meet the minimum nucleic acid or sequencing quality standards and were excluded from the analysis. This reduction in sample size may have obscured subtle changes in the resistome or microbiome and limited our ability to perform paired-sample comparisons. Fourth, we did not have data on the timing of the doxy-PEP doses with respect to the rectal swab sample collection or longer-term follow-up samples to determine time to normalization of the microbiome and resistome. Fifth, we only evaluated the gut microbiome, and doxy-PEP use may impact other microbiome sites, such as skin and nasopharynx, differently. Sixth, our specimens are limited to 6 months of follow-up; longer-term data are needed to understand the impact of doxy-PEP with more extended use. Finally, as we used short-read Illumina sequencing, we were unable to definitively link tetracycline ARGs to specific bacterial species and, thus, had to rely on abundance correlation analyses as a proxy.

In sum, we found that doxy-PEP use increased both the relative proportion and expression of tetracycline ARGs while minimally impacting the ecology of the gut microbiome. These findings contribute to understanding of the ecological impacts of doxy-PEP on the human gut microbiome and antimicrobial resistome, which, at baseline, are enriched in tetracycline ARGs. Further investigations are needed to explore the clinical implications of our findings, including population-based surveillance to monitor for emergence of tetracycline-resistant pathogens as doxy-PEP is more widely implemented in eligible populations.

## Methods

### Study design, clinical cohort and ethics statement

The DoxyPEP trial (ClinicalTrials.gov registration number: NCT03980223)^2^ compared doxy-PEP use (doxycycline post-exposure prophylaxis) to standard of care (no post-exposure prophylaxis) for 501 participants. The study was conducted at two HIV clinics and two sexual health clinics in San Francisco and Seattle. Individuals were eligible for enrollment if they were at least 18 years of age; had male sex assigned at birth; had received a diagnosis of HIV or were on HIV PrEP; and had received a bacterial STI diagnosis of gonorrhea, chlamydia or early syphilis in the previous 12 months. Participants were randomized in a 2:1 ratio to the DP arm or the SOC arm. Participants in the DP arm were counseled to take a 200-mg doxycycline hyclate dose within 72 h after condomless anogenital, vaginal or oral sex and no more than one dose every 24 h. Participants in both arms self-collected rectal swabs at enrollment (day 0) and at a 6-month visit (month 6). Demographic and clinical information (for example, age, HIV infection status, number of doxy-PEP doses and antibiotic exposures during the study period) were collected for each participant via RedCAP^39,40^ (hosted at the University of Washington) and Microsoft Excel. Among all 501 participants in the DoxyPEP trial, 292 (58%) had chlamydial infection in the prior year and were likely to have taken a course of doxycycline for chlamydia treatment at least once in the prior year. Additional information on doxycycline exposure before enrollment and timing of the doxy-PEP use before sample collection was unavailable. The study protocol^2^ was approved by the University of California, San Francisco institutional review board, which served as the primary institutional review board. All participants provided written informed consent, and no monetary compensation was provided for participation.

For this analysis, a subset of 150 participants from the 510 DoxyPEP trial participants was selected for metagenomic sequencing of self-collected rectal swab samples. The 150 participants were selected based on the following criteria: (1) study arm group (50 SOC, 100 DP); (2) HIV infection status (1:1 of participants living with HIV and participants on HIV PrEP); and (3) availability of both day-0 and month-6 rectal samples (Fig. 1). The SOC participants were a simple random sample, whereas the DP participants were the top 50 participants, including participants both with and without HIV infection, with the highest reported combined doxy-PEP use on the month-3 and month-6 study visits.

### Cohort description

We performed descriptive analysis of participant demographics and compared the participants in the DP arm to participants in the SOC arm. No sex or gender analysis was carried out given that the study population enrolled only those who were assigned male sex at birth. *P* values for categorical variables were obtained using the Pearson’s chi-square test and Fisher’s exact test if counts were less than 5; *P* values for continuous variables were calculated using the two-sided Wilcoxon rank-sum test.

### Metagenomic sequencing

Metagenomic sequencing of DNA-seq and RNA-seq was performed on the day-0 and month-6 rectal swabs from the 150 participants. Swabs were self-collected into DNA/RNA Shield collection tubes (Zymo Research, R1107-E) and stored at −80 °C within 2 weeks of collection. Total nucleic acid was extracted from 500 µl of DNA/RNA Shield solution using a previously described modified cetyltrimethylammonium bromide (CTAB)-based protocol^41^ and in samples with sufficient yield, normalized to 10 ng of total input per sample.

DNA-seq was carried out using a NEBNext Ultra II DNA Kit (New England Biolabs, E7645L). Before RNA-seq, human cytosolic and mitochondrial ribosomal RNA was depleted using FastSelect (Qiagen, 334385). RNA was then fragmented and underwent library preparation using a NEBNext Ultra II RNA-seq Kit (New England Biolabs, E7770L) according to the manufacturer’s instructions. Both DNA-seq and RNA-seq library preparation protocols were optimized for a LabCyte Echo acoustic liquid handler^42^. Finished libraries underwent paired-end Illumina sequencing on a NovaSeq 6000 instrument.

For the purposes of background contamination correction and to enable estimation of microbial mass, negative water controls and positive controls (spike-in RNA standards from the External RNA Controls Consortium (ERCC), Thermo Fisher Scientific, 4456740)^43^ were included in every RNA sample before RNA-seq library preparation. Reverse-transcribed complementary DNA ERCC standards were spiked into every DNA sample before DNA-seq library preparation.

### Detection of microbes and ARGs

We leveraged the open-source CZ ID pipeline (https://czid.org/) as a first step to detect both microbes (mNGS pipeline version 8.1) and ARGs (AMR pipeline version 1.2.15)^44^. For microbial detection, the CZ ID pipeline performed subtractive alignment of the human genome (National Center for Biotechnology Information (NCBI)) from input raw FASTQ files, followed by quality and complexity filtering. The remaining microbial reads were then identified by an assembly-based alignment against reference genomes from the NCBI nucleotide (NT) database. After background correction (see below), all remaining taxa with at least 10 hits to the NCBI NT database and one hit to the NCBI non-redundant (NR) protein database with a minimum alignment length of 50 bases were retained for downstream microbiome analyses. All samples with more than 100,000 reads and, for DNA-seq samples, samples with a duplicate compression ratio less than 10 were retained for downstream analyses of microbes and ARGs. CZ ID’s antimicrobial resistance pipeline implements the Comprehensive Antibiotic Resistance Database (CARD)^45,46^ Resistance Gene Identifier (RGI) tool, which aligns quality-controlled reads against the CARD databases (canonical CARD version 3.2.6 and WildCARD version 4.0.0) of ARG sequences. ARGs with ≥5% read coverage breadth were retained for downstream analyses.

### Identification and mitigation of environmental contaminants

Negative water controls were processed in parallel with the participant samples for microbial and ARG detection, allowing for an estimation of the number of background reads expected for each taxon and ARG^42^. A negative binomial model was used to identify and select for taxa and ARGs present in the participant samples at an abundance significantly greater than in the negative controls^47^. The number of background reads was modeled as a negative binomial distribution, with mean and dispersion fitted on the negative controls. For each batch (DNA-seq only) and taxon/ARG, the mean parameter of the negative binomial was estimated by averaging the read counts across all negative controls. Using the functions glm.nb() and theta.md() from the R package MASS^48^ (version 7.3.58.1), a single dispersion parameter across all taxa was then estimated. Taxa associated with *P* ≥ 0.05 were excluded; *P* values were adjusted for multiple comparisons using the Benjamini–Hochberg false discovery rate (FDR) method.

### Mass calculations

Microbial mass and ARG mass were calculated based on the total reads aligning to the ERCC RNA standards^43^ spiked into each sample (RNA-seq) or reverse-transcribed cDNA ERCC standards (DNA-seq). ERCC input mass was 25 pg for DNA-seq samples and 2.5 pg for RNA-seq samples. The following equations were used for microbial input mass, normalized by total million sequencing reads to account for sample variation in input mass:

$${\rm{microbial\; input\; mass}}=\frac{\frac{{\rm{microbial\; reads}}* {\rm{ERCC\; input\; mass}}}{{\rm{ERCC\; reads}}}}{{\rm{sequencing\; reads}}({\rm{millions}})},$$

and, for ARG input mass, normalized by total million sequencing reads:

$${\rm{ARG\; input\; mass}}=\frac{\frac{{\rm{ARG\; depth}}* {\rm{ERCC\; input\; mass}}}{{\rm{ERCC\; reads}}}}{{\rm{sequencing\; reads}}({\rm{millions}})}.$$

ARG depth was defined as the mean read depth across the references sequence. The mass of an ARG class was the summation of the mass of all ARGs belonging to the class of interest. Similarly, total microbial or ARG mass of each sample was a summation of the mass of all microbes or ARGs, respectively.

### Statistical analyses

#### Resistome analysis

We evaluated the impact of doxy-PEP use on ecological parameters, including the resistome alpha diversity, resistome beta diversity and log_10_-transformed total resistome mass. Alpha diversity was calculated by Shannon diversity index, accounting for ARG abundance (depth per million (dpm)) and evenness. Beta diversity among samples with at least one ARG was calculated using Bray–Curtis dissimilarity with 1,000 permutations, accounting for presence/absence and abundance of the ARGs (dpm). Analysis of multivariate homogeneity of group dispersions was performed using the functions betadisper() and permutest(). Beta diversity was displayed via non-metric multi-dimensional scaling (NMDS) and the function metaMDS(). One outlier from the DP arm was omitted from the RNA-seq beta diversity plot for graphical purposes (coordinates NMDS1: 9.9; NMDS2: −0.3) but was included in the calculations. The adonis2() function was used to perform a PERMANOVA test and adjusted for multiple comparisons. Both diversity calculations were performed using the R package ‘vegan’ (version 2.6.4)^49^.

We assessed the impact of doxy-PEP use on tetracycline ARG richness (number of distinct ARG types) and proportion of each ARG class mass to the total resistome mass. We focused on ARG classes where the median proportion of the ARG class mass of the resistome mass per sample was more than 1% in any of the following subgroups (SOC day-0, SOC month-6, doxy-PEP day-0 and doxy-PEP month-6) for DNA-seq or RNA-seq data; these ARG classes included aminoglycosides, beta-lactams, MLS, sulfonamide/trimethoprim and tetracyclines (Supplementary Table 2). ARGs that included tetracycline resistance but also conferred resistance to multiple other classes were ‘multi-drug efflux pumps’; these were not included in the ARG class analysis given that the proportional mass was less than 1% of the resistome mass (Supplementary Table 2). We also compared ARG class abundance and expression; both were measured and normalized per million reads sequenced and gene length (dpm) in the metagenome and the metatranscriptome, respectively. Within the tetracycline ARGs, we described the different mechanisms of resistance (tetracycline target protection, tetracycline inactivation and tetracycline-specific efflux pumps) detected.

We used inflated beta-regression models to examine the association between doxy-PEP use (independent variable) and the proportional ARG class mass within the resistome (dependent variable) using the function gamlss() (family = ’BEINF’) from the R package ‘gamlss’ (version 5.4-22). We included HIV infection status and the number of days of cephalosporin exposures between enrollment and sample collection as covariates in the inflated beta regression models. We chose to include only the cephalosporin exposure days within the models as exposure to other classes of antibiotics was limited and not found to be significant between the SOC and DP arms.

We evaluated whether there was a dose-dependent relationship between the number of reported doxycycline doses taken since enrollment and changes in the resistome. We considered a prophylactic dose (doxycycline 200 mg one time) as a single dose. For patients receiving doxycycline for STI treatment (doxycycline 100 mg twice a day for 7 d), we considered a treatment day to be equivalent to a single prophylactic dose. The number of doxycycline doses was categorized as follows: 0 doses, 1–25 doses, 26–50 doses and ≥50 doses. These categories were chosen based on the distribution of the number of doxycycline dose exposures among the participants studied and in consideration of the median number of doxy-PEP doses (24 doses) taken over 6 months by the 339 DP arm participants in the DoxyPEP clinical trial^2^. Spearman’s *ρ* test of trend (cor.test) from the R package ‘stats’ (version 4.2.1) was performed across these ordinal doxycycline dose categories for tetracycline ARG richness and proportion of tetracycline ARG to the resistome mass. In addition, we evaluated the association between doxycycline dose exposure as a continuous variable with the two separate outcomes of tetracycline ARG richness and proportional tetracycline ARG mass using linear regression models for tetracycline ARG richness and inflated beta regression models for proportional tetracycline ARG mass.

A sub-analysis of paired samples was performed to evaluate the impact of doxy-PEP use on tetracycline ARG richness and tetracycline ARG proportion of the resistome mass. *P* values were calculated using the two-sided Wilcoxon signed-rank test for paired samples (wilcox_test, paired = TRUE) from the R package ‘rstatix’ (version 0.7.2). For all non-paired comparison tests, *P* values were obtained by the two-sided Wilcoxon rank-sum test (wilcox_test, paired = FALSE).

### Microbiome analysis

To examine the effect of doxy-PEP use on the global microbiome taxonomic composition, we analyzed the normalized and transformed mass of the bacterial components of the microbiome. We also examined differences in diversity metrics of the microbiome between the two arms at both timepoints and between timepoints within arms. Bacterial alpha diversity was calculated using the Shannon diversity index, accounting for bacterial abundance (nucleotide reads per million (nt rpm)) and evenness. Bacterial beta diversity was calculated using Bray–Curtis dissimilarity in a similar manner to the resistome analysis, substituting bacterial abundance by nt rpm, with the R package ‘vegan’ (version 2.6.4)^49^. To examine microbiome changes at the genus level, we performed differential abundance analyses, adjusted for multiple comparisons, using a consensus approach of three differential abundance methods to ensure a robust biological interpretation. We used the R packages ‘DESeq2’ (version 1.36.0), ‘metagenomeSeq’ (version 1.40.0) and ‘ALDEx2’ (version 1.30.0). In the DESeq2 analysis, a pseudo-count (one read) was added to all taxa counts to address zero inflation of microbiome data. Specific species of interest, including common sexually transmitted organisms, were also analyzed for differential abundance between day 0 and month 6 in the DP arm using the two-sided Wilcoxon rank-sum test.

### Microbiome and ARG correlation

To identify microbial taxa associated with tetracycline ARGs, Spearman’s correlation analyses were performed using the functions cor() and cor_pmat() from the R package ‘rstatix’ (version 0.7.2) on paired day-0 and month-6 samples from the DP arm. The correlation analyses were between the abundance (DNA-seq) or expression (RNA-seq) of tetracycline ARGs (dpm) and microbial taxa (rpm). Correlation analyses were adjusted for multiple comparisons. These analyses were performed at the genus level, comparing the 50 most abundant bacterial taxa in combination with tetracycline resistance genes. For correlations that were statistically significant at month 6 between tetracycline ARGs and bacterial taxa, we evaluated the change in the strength of correlations over time with doxy-PEP use by calculating the difference in the SCC from day 0 to month 6 (ΔSCC = SCC_month6_ − SCC_day0_).

All analyses were conducted in RStudio (version 2023.09.1+494) using R (version 4.2.1) and performed for both DNA-seq and RNA-seq data. All adjustments for multiple comparisons were by the Benjamini–Hochberg FDR method. Figures were made using the following R packages: ‘ggplot2’ (version 3.5.1) and ‘scales’ (version 1.3.0).

### Reporting summary

Further information on research design is available in the Nature Portfolio Reporting Summary linked to this article.

## Online content

Any methods, additional references, Nature Portfolio reporting summaries, source data, extended data, supplementary information, acknowledgements, peer review information; details of author contributions and competing interests; and statements of data and code availability are available at 10.1038/s41591-024-03274-2.

## Supplementary information

Supplementary Information Supplementary Tables 1 and 2 and DoxyPEP Study Protocol.

Reporting Summary

## Data Availability

FASTQ files containing non-host reads identified by the CZ-ID pipeline, after subtraction of reads aligning to the human genome, are available from the National Center for Biotechnology Informationʼs Sequence Read Archive under BioProject ID PRJNA1099775. De-identified patient data and source data can be found at https://github.com/infectiousdisease-langelier-lab/doxy-PEP.

## Code availability

All code can be found at https://github.com/infectiousdisease-langelier-lab/doxy-PEP.
