# Supplementary material for: Impact of doxycycline post-exposure prophylaxis for sexually transmitted infections on the gut microbiome and antimicrobial resistome
Source: Nat Med. 2024 Oct 3;31(1):207–17. doi: 10.1038/s41591-024-03274-2 (PMC11750720; doi:10.1038/s41591-024-03274-2)
Supplement: Supplementary file 1 — Supplementary Tables 1 and 2 and DoxyPEP Study Protocol. [file 41591_2024_3274_MOESM1_ESM.pdf]

nature medicine

Supplementary information https://doi.org/10.1038/s41591-024-03274-2

Impact of doxycycline post-exposure prophylaxis for sexually transmitted infections on the gut microbiome and antimicrobial resistome

In the format provided by the authors and unedited

Supplementary Table 1. Inflated beta-regression models of the proportion of the resistome mass, by antimicrobial resistance gene (ARG) class for participants from the doxy-PEP month-6 arm compared with doxy-PEP day-0 arm (reference group) by (a) DNA-seq and (b) RNA-seq. The model included HIV infection status and cephalosporin exposure (defined as number of cephalosporin treatment days received since enrollment at the time of sample collection) as covariates. Abbreviation: MLS, macrolide-lincosamide-streptogramin.

a

**DNA-seq (n = 89 participants)**		
**ARG class**	**μ estimate (95% confidence interval)**	**p-value (two-sided Wald's test)**
Aminoglycoside	-0.25 (-0.56 – 0.06)	0.12
Beta-lactam	-0.14 (-0.35 – 0.08)	0.22
MLS	-0.23 (-0.48 – 0.25)	8.2×10^{-2}
Sulfonamide/Trimethoprim	0.12 (-0.19 – 0.44)	0.45
Tetracycline	0.45 (0.19 – 0.70)	9.2×10^{-4}

b

**RNA-seq (n = 70 participants)**		
**ARG class**	**μ estimate (95% confidence interval)**	**p-value (two-sided Wald's test)**
Aminoglycoside	-0.06 (-1.13 – 1.02)	0.92
Beta-lactam	-0.20 (-0.79 – 0.39)	0.51
MLS	-0.76 (-1.37 – -0.15)	1.9×10^{-2}
Sulfonamide/Trimethoprim	-0.09 (-1.25 – 1.07)	0.88
Tetracycline	1.5 (0.94 – 2.00)	1.7×10^{-6}

Supplementary Table 2. The proportional mass of the resistome mass by antimicrobial resistance gene (ARG) classes by (a) DNA-seq and (b) RNA-seq.

a

**DNA-seq (n = 127 samples)**				
**ARG class**	**Median proportional mass of resistome, by ARG class (%)**			
	**SOC Day 0 n = 21**	**SOC Month 6 n = 26**	**Doxy-PEP Day 0 n = 37**	**Doxy-PEP Month 6 n = 43**
Aminoglycoside	5%	4%	4%	3%
Beta-lactam	16%	13%	15%	13%
Chloramphenicol	0.4%	0.4%	0.4%	0.4%
Fluoroquinolone	0.2%	0.2%	0.2%	0.2%
Glycopeptide	0%	0%	0%	0%
MLS	21%	22%	24%	19%
Multi-drug efflux pump	0.1%	0.8%	0.2%	0.5%
Peptide	0%	1%	0%	0.1%
Sulfonamide/Trimethoprim	2%	3%	2%	3%
Tetracycline	45%	44%	47%	53%
Miscellaneous	1%	1%	1%	0.8%

b

**RNA-seq (n = 86 samples)**				
**ARG class**	**Median proportional mass of resistome, by ARG class (%)**			
	**SOC Day 0 n = 9**	**SOC Month 6 n = 22**	**Doxy-PEP Day 0 n = 17**	**Doxy-PEP Month 6 n = 38**
Aminoglycoside	0%	0%	0%	0%
Beta-lactam	58%	30%	46%	49%
Chloramphenicol	0%	0%	0%	0%
Fluoroquinolone	0%	0%	0%	0%
Glycopeptide	0%	0%	0%	0%
MLS	15%	28%	40%	13%
Multi-drug efflux pump	0%	0%	0%	0%
Peptide	0%	0%	0%	0%
Sulfonamide/Trimethoprim	0%	0%	0%	0%
Tetracycline	7%	5%	3%	18%
Miscellaneous	0%	0%	0%	0%

PROTOCOL

DoxyPEP: Evaluation of doxycycline post-exposure prophylaxis to reduce sexually transmitted infections in men who have sex with men and transgender women either on PrEP or living with HIV

Version 9.0

May 27, 2022

TABLE OF CONTENTS

STUDY TEAM....4
GLOSSARY....5
SCHEMA....6
1 BACKGROUND AND SIGNIFICANCE....8
1.1 Background....8
1.2 Rationale....10
2 STUDY DESIGN....13
3 OBJECTIVES....14
3.1 Primary Objectives....14
3.2 Secondary Objectives....14
3.3 Exploratory objectives....14
4 SELECTION AND ENROLLMENT OF PARTICIPANTS....14
4.1 Inclusion criteria....15
4.2 Exclusion criteria....15
4.3 Recruitment....15
4.4 Co-enrollment guidelines....16
5 STUDY TREATMENT....16
5.1 Study product....16
5.2 Safety of doxycycline....16
5.3 Doxycycline dispensing and administration....17
5.4 Concomitant medications....17
5.5 HIV pre-exposure prophylaxis (PrEP) and HIV antiretroviral therapy (ART)....17
6 CLINICAL AND LABORATORY MONITORING....18
6.1 Schedule of Events....18
6.2 Study visits....20
6.3 Instructions for evaluations....20
6.4 Evaluation at time of presumptive or diagnosed GC, CT and syphilis....23
6.5 Testing for resistance in setting of STI diagnoses on study....25
6.6 Treatment of STIs....26
6.7 Qualitative interviews....26
6.8 Reenrollment of participants enrolled prior to 3/15/2020....26
7 ADVERSE EVENTS (AE) AND STUDY MONITORING....26
7.1 Adverse Event Collection Requirements....26
7.2 AE and SAE attribution to doxycycline....27
7.3 Study Monitoring....27
8 CLINICAL MANAGEMENT ISSUES....28
8.1 Toxicity....28

8.2 Specific Management of Toxicities Related to Study-Provided Drugs 28
9 CRITERIA FOR DISCONTINUATION 29
9.1 Premature Study Treatment Discontinuation 29
9.2 Premature Study Discontinuation 29
10 STATISTICAL CONSIDERATIONS 29
10.1 Outcome measures 29
10.2 Statistical power and analysis 30
10.3 Endpoint adjudication 31
10.4 Post COVID follow-up 32
10.5 Analyses post 5/13/2022 closure to enrollment 32
11 PARTICIPANTS 32
11.1 Institutional Review Board (IRB) Review 32
11.2 Informed Consent 32
11.3 Study records 32
11.4 Confidentiality 33
12 BIOHAZARD CONTAINMENT 33
REFERENCES 34

STUDY TEAM

Connie Celum, MD, MPH, co-Principal Investigator
Annie Luetkemeyer, MD, co-Principal Investigator
Jared Baeten, MD, PhD
Ruanne Barnabas, MBChB, DPhil
Susan Buchbinder, MD, MPH
Katerina Christopoulos, MD, MPH
Edwin D. Charlebois, MPH, PhD
Stephanie Cohen, MD, MPH
Julie Dombrowski, MD, MPH
Deborah Donnell, PhD
Rob Fredericksen, PhD
Diane Havlir, MD
Chaz Langelier, MD,PhD
Hyman Scott, MD, MPH
Olusegun Soge, MSc, PhD
Vivek Jain, MD, MAS
Emma Bainbridge, MD, MPH

GLOSSARY

AE	Adverse event
AMR	Antimicrobial resistance
ART	Antiretroviral therapy
CT	*Chlamydia trachomatis*
GC	Gonorrea
HIV	Human Immunodeficiency virus
LGV	Lymphogranuloma venereum
MG	Mycoplasma genitalium
MSM	Men who have sex with men
NAAT	Nucleic acid amplification test
NGU	Non-gonococcal urethritis
PEP	Post-exposture prophylaxis
PLWH	People living with HIV
PrEP	Pre-exposure prophylaxis
RPR	Rapid Plasma Reagin
SAE	Serious Adverse Event
STI	Sexually transmitted infection
TCN	Tetracycline
TGW	Transgender women
VDRL	Venereral Disease Research Lab

SCHEMA

DoxyPEP: Prospective, randomized, open-label, clinical trial to evaluate the impact of doxycycline post-exposure prophylaxis on the occurrence of bacterial STIs among men who have sex with men (MSM) and transgender women (TGW) PrEP users and people living with HIV

Design: Open-label randomized clinical trial of doxycyline PEP to reduce bacterial STIs (*N. gonorrheae* [GC], *C. trachomatis* [CT], and *T. pallidum* [syphilis]) among MSM and transgender women (TGW) living with HIV (PLWH) or on HIV PrEP.

Study Population: Sample size of 780, approximately equal numbers of PLWH and participants taking HIV pre-exposure prophylaxis (PrEP)

Study Sites:

1. HIV/AIDS Clinic ("Ward 86"), Zuckerberg San Francisco General Hospital, UCSF, San Francisco
2. San Francisco City Clinic, San Francisco Department of Public Health, San Francisco (municipal STD clinic)
3. Madison HIV/AIDS Clinic, Harborview Medical Center University of Washington, Seattle
4. Public Health-Seattle & King County Sexual Health Clinic at Harborview Medical Center, Seattle

Primary Study Objectives:

1. Evaluate the effectiveness of doxycycline post-exposure prophylaxis (PEP) to reduce STI infections in two populations: a) MSM living with HIV and b) MSM/TGW taking HIV PrEP
2. Investigate the impact of doxycycline PEP on development of culture-based tetracycline (TCN) resistance in *N. gonorrheae* and among nasopharyngeal carriers of *S. aureus*

Secondary Study Objectives:

1. Assess the safety, tolerability, and acceptability of doxycycline PEP
2. Investigate the impact of doxycycline PEP on detection of culture and molecular markers of tetracycline (TCN) resistance in *C. trachomatis* (CT*), and T. pallidum* (syphilis- molecular markers only)
3. Evaluate the development of phenotypic tetracycline resistance among oropharyngeal carriers of commensal *Neisseria* species
4. Measure the proportion of sex acts which are covered by doxycycline PEP
5. Assess the effect of doxycycline PEP on the gut resistome through measuring the abundance of tetracycline resistance genes in stool of a subset of 50 MSM living with HIV and 50 MSM/TGW on PrEP assigned to receive doxycycline PEP as well as rectal swabs from all participants, stored for future evaluation

Exploratory Objectives:

1. Evaluate the diversity of the gut microbiome and quantity and breadth of drug resistance genes in participants on doxycycline PEP and not on PEP
2. Assess the association between meningococcal vaccination and incident GC infection
3. Evaluate the incidence of nongonococcal, nonchlamydial urethritis and the proportion of urethritis in which *Mycoplasma genitalium* (MG) is detected
4. Assess the rate of detection of *Mycoplasma genitalium* (MG) in urine in each arm and the presence of tetracycline and other antimicrobial resistance in MG in each arm.

5. Evaluate association of doxycycline use with weight change over time
6. Evaluate whether doxycycline PEP is associated with PrEP persistence and HIV virologic suppression by comparison of arms and within the doxycycline PEP arm by adherence to doxycycline PEP.
7. Evaluate the incidence of lymphogranuloma venereum (LGV) in those diagnosed with rectal chlamydia in each arm

Approach: Eligible participants randomized to receive PEP will receive open-label doxycycline 200 mg to be taken ideally within 24 hours but no later than 72 hours after each condomless sexual contact (receptive anal, insertive anal, vaginal, or oral, or use of shared sex toys). No more than 200 mg will be taken in each 24 hour period. Sexual activity and doxycycline PEP usage will be recorded using a smartphone application and assessment at study visits. At enrollment and quarterly for 12 months of follow-up, GC and CT testing will be conducted on samples from the oropharynx, rectum and urine and a blood specimen for syphilis serology will be collected. Resistance testing will be conducted on specimens that test positive for GC using phenotypic methods, and molecular techniques for CT and syphilis (for syphilis, DNA obtained from mucosal or lesional swabs). Nares and pharyngeal swabs will be obtained to evaluate tetracycline resistance among nasopharyngeal carriers of *S. aureus* and commensal *Neisseria* species (oropharyngeal swab only). Stool samples from 50 MSM/TGW living with HIV and 50 MSM/TGW on PrEP in the doxycycline PEP arm at 3 time points will undergo metagenomic sequencing for tetracycline resistance genes. Rectal swabs from all participants who consent will be archived for future evaluation of the enteric microbiome and resistome, as well as for presence of MG.

Study Schema

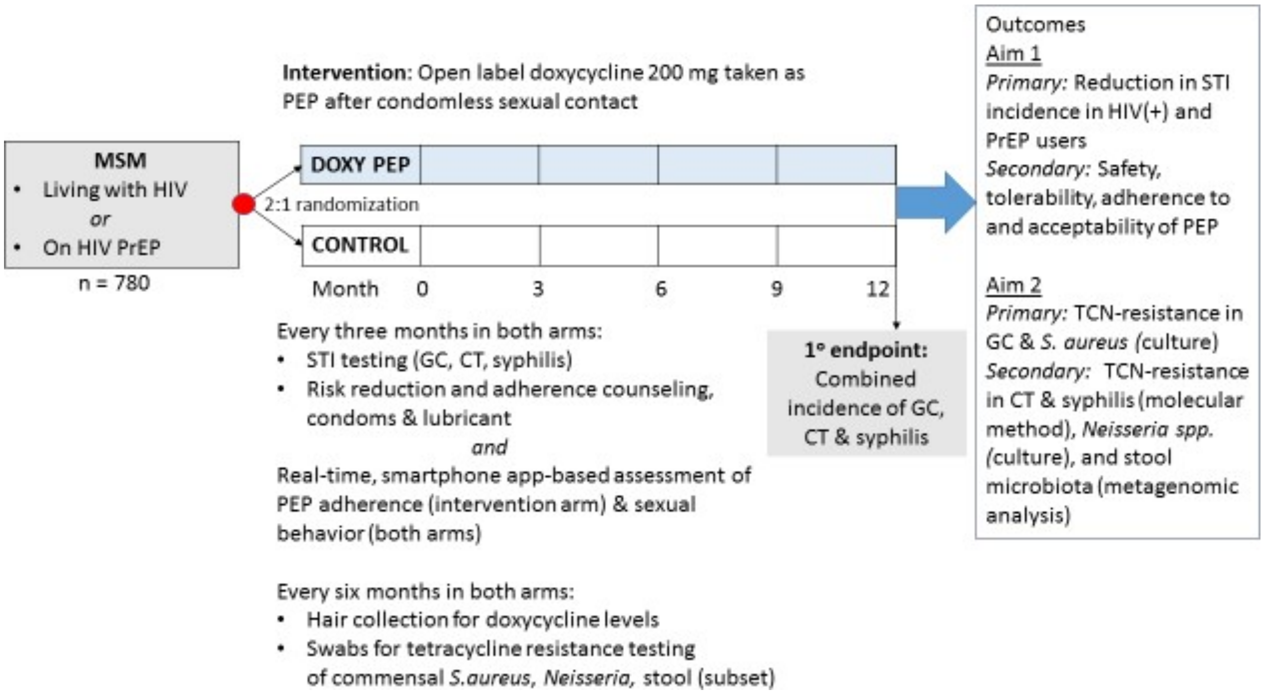

1 BACKGROUND AND SIGNIFICANCE

1.1 Background

The incidence of bacterial sexually transmitted infection (STI) continues to increase in the US, with more than 2 million diagnoses of syphilis, gonorrhea (GC) and chlamydia (CT) in 2016.[2] This STI epidemic is occurring globally and is concentrated among men who have sex with men (MSM) and transgender women (TGW), including those living with and without HIV. In the US, MSM accounted for 81% of syphilis infections in 2016 and 38% of gonorrhea cases; the latter represents a nearly 10-fold increase since 1989. [2] CT is also increasing among MSM, with rectal chlamydia rates of 17% and the highest rates of CT diagnosed in STI clinics in MSM ≥ 25 years old.[2] Both MSM living with HIV and MSM without HIV infection on PrEP have very high bacterial STI incidence, reaching or exceeding 50% new STI diagnoses per year.[3, 4]

Increasing STI rates have been associated with a rise in serious morbidity, including blindness secondary to syphilitic ocular complications.[5] Congenital syphilis rates have increased 28% in the past few years;[6] it is likely that some of this increase comes from 'bridging' from MSM to heterosexual populations.[7] Distressingly, drug-resistant GC continues to rise globally[8-10] and in the US,[11] leading to limited oral treatment options; recent cases of highly-resistant GC in the UK and Australia required treatment with ertapenem.[12, 13] Untreated STIs increase the risk of HIV transmission from individuals with HIV infection who are not virologically suppressed,[14, 15] as occurs in nearly half of those living with HIV nationwide.[16] With the recent CDC-endorsed "U=U" campaign (i.e., undetectable=untransmittable),[17] condom use may further decrease among persons with HIV infection, and may impact 'serosorting' in selection of sexual partners and decisions to use condoms. Accordingly, there is an urgent need for innovative, effective, and acceptable interventions to halt the rise in syphilis, GC, and CT among MSM and TGW living with and at-risk for HIV infection.

One potential STI control strategy for these high-risk populations is post-exposure prophylaxis (PEP) with doxycycline. Antibiotic prophylaxis against STIs is not a new concept; minocycline was used as PEP for GC in the US Navy in the 1940s and showed transient benefits with no clinical harms, but the rapid emergence of tetracycline resistance prevented uptake of this approach as a public health strategy [18] A systematic review of presumptive periodic treatment of sex workers showed overall 50% reductions in GC and CT without risk compensation or increased AMR, and this approach is recommended for populations with high baseline GC and CT prevalence [19]. The rationale for STI prophylaxis among MSM and TGW is similar, given the high prevalence and incidence of bacterial STIs among MSM living with HIV and MSM on PrEP. An effective STI prophylaxis strategy could augment frequent STI screening, partner notification and other efforts to reduce STI acquisition and transmission among MSM.

Doxycycline has good tolerability, high bioavailability with oral dosing, a 20 hour half-life, and excellent tissue penetration. Doxycycline is a first line treatment for CT with very high efficacy (97-100%). Doxycycline is used to treat syphilis for persons with penicillin allergy. Notably, CT and syphilis have not developed TCN resistance in spite of decades of doxycycline use to treat these infections. Doxycycline is not recommended for GC treatment due to baseline resistance and increased TCN resistance will not affect GC management.

Figure 1. IPERGAY Study: Time to first STI (panel a), GC infection (panel b), CT infection (panel c), and syphilis infection (panel d) [1]

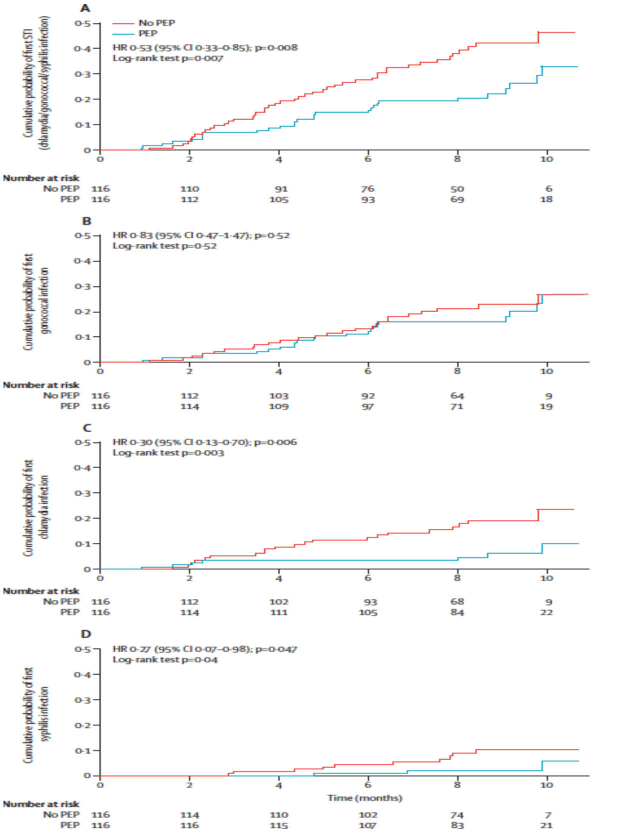

The IPERGAY study demonstrated efficacy of single dose doxycycline PEP after condomless sex among 232 HIV-negative MSM taking event-driven PrEP in France.[20] Doxycycline PEP resulted in a 47% relative reduction in new bacterial STIs (GC, CT, or syphilis) over 8.7 months of follow-up (45.9 vs. 79.6 per 100 person-years in those assigned to doxycycline PEP vs. no PEP control, respectively; Figure 1A. Doxycycline PEP reduced the incidence of CT and syphilis, but not GC (Figure 1B-D), which was not surprising given doxycycline resistance in >50% of GC strains in France. MSM in IPERGAY had a median age of 38 years, were highly educated, majority Caucasian, and highly adherent to event-driven HIV PrEP. MSM in the doxycycline arm in IPERGAY took a median of 680 mg of doxycycline per month. Adverse events were rare and there was no difference in self-reported sexual behavior between MSM in the two study arms.

Limitations of generalizability from the IPERGAY study include that the sample was small (n=232), and in contrast to MSM in the US heavily impacted by STIs, MSM were older (median age 38), highly educated, and majority Caucasian. In addition, with respect to adherence to event-driven doxycycline PEP, MSM in the IPERGAY study were highly adherent to event-driven HIV PrEP and were already 'cueing' their TDF-FTC (Truvada) PrEP dosing to their sexual activity. Importantly, uptake and adherence to doxycycline PEP among daily PrEP users are unknown. Additionally, IPERGAY did not conduct detailed measurements of the pattern of doxycycline use with sexual practices nor evaluate for antibiotic resistance associated with intermittent doxycycline use. Doxycycline PEP may be more efficacious for reducing GC incidence in the US than in Europe, due to lower GC TCN resistance in the US (25% vs 55% respectively).[11, 20] PEP may also be more effective to prevent GC at non-pharyngeal sites, as evidenced by the lack of an observed effect on pharyngeal GC incidence, whereas there was a trend toward reduction in rectal and urethral GC infections with doxycycline PEP in IPERGAY.[21]

A small study piloted daily doxycycline among 30 MSM living with HIV with a history of syphilis, and showed a 73% reduction in composite STIs, without power to assess individual STIs.[22] The greater risk of side effects and antimicrobial resistance (AMR) with daily dosing of doxycycline have dampened enthusiasm for daily dosing of doxycycline as PrEP against STIs. Event-driven doxycycline PEP has not been studied in MSM living with HIV.

Significant scientific knowledge gaps remain about on-demand STI doxycycline PEP in PrEP users and MSM living with HIV. MSM in IPERGAY were already 'cueing' their Truvada PrEP dosing to sexual activity; PEP adherence in daily PrEP users is unknown. IPERGAY did not conduct detailed measurements of the pattern of doxycycline use with sexual practices nor report antibiotic resistance associated with intermittent doxycycline use. Doxycycline PEP may be more efficacious for reducing GC incidence in the US than in Europe, due to lower TCN resistance in GC the US (30% vs 55% respectively).[11, 20] PEP may also be more effective to prevent GC at non-pharyngeal sites; there was a trend toward reduction in rectal and urethral infections with doxycycline PEP in IPERGAY.[21] Notably, based on the IPERGAY demonstration of efficacy with doxycycline PEP, providers have

begun to prescribe doxycycline PEP to reduce incident STIs in MSM without a confirmatory study or normative guidance. Moreover, the balance of benefits and risks of doxycycline PEP need to be carefully studied in MSM living with HIV as well as in PrEP users in a study powered to assess effectiveness for each population. The relative benefits and risks of doxycycline PEP may differ in MSM living with HIV and PrEP users due to differences in adherence, sexual practices, potential risk compensation, sexual networks, and/or pre-existing antibiotic resistance due to higher prior antibiotic exposure among MSM living with HIV.

In summary, a sufficiently-powered, high-quality study is needed to evaluate the effectiveness of doxycycline PEP in populations both with and without HIV infection, and to evaluate its impact on drug resistance in target bacterial STIs and resident bacterial microbiota. There is a public health need and sufficient clinical equipoise to conduct an effectiveness study of doxycycline PEP as an STI reduction strategy for a diverse population of MSM in the US to address the following key scientific questions: 1) *Effectiveness of doxycycline PEP for bacterial STI prevention in MSM and TGW on PrEP,* 2) *Effectiveness of doxycycline PEP in MSM and TGW living with HIV;* 3) *Differential tolerability and impact on antimicrobial resistance between MSM/TGW living with HIV and MSM/TGW on PrEP; 4) TCN resistance in GC, CT and syphilis, 5) TCN resistance in commensal Neisseria (which can transfer TCN resistance) and potentially pathogenic Staph Aureus in carriers, and 6) effect on the gut resistome in terms of changes in TCN resistance genes*.

1.2 Rationale

The overall goal of this study is to understand the effectiveness of doxycycline PEP on reducing STIs in MSM and TGW populations at high risk for STI acquisition and to evaluate its impact on antibiotic resistance.

1.2.1 Doxycycline Safety

Several features of doxycycline make it an optimal choice for STI PEP. Doxycycline has been used safely and for extended durations as prophylaxis against infections such as malaria[23] and for non-infectious conditions including rosacea[24] and acne vulgaris.[25] Doxycycline is generally safe and well-tolerated when used chronically. Side effects include mild gastrointestinal symptoms, photosensitivity, and rarely pill esophagitis and pseudotumor cerebri.[26] Reassuringly, doxycycline has a much lower risk than other antibiotics for the antibiotic-associated diarrhea caused by *C. difficile* infection,[27, 28] and may even exert a protective effect against *C. difficile.*[29, 30] Doxycycline is a scalable PEP intervention, as it is generic, inexpensive, and widely available. Doxycycline does not require dose adjustment in hepatic or renal failure, including dialysis. In the IPERGAY doxycycline PEP study, there was no difference in grade 3 or 4 adverse events between the study arms. As anticipated, there were more gastrointestinal side effects in those on PEP (25%) vs. the non-PEP control arm (14%). Discontinuation of doxycycline was uncommon with 8 study participants (7%) stopping doxycycyline due to side effects attributed to doxycycline, the majority of which were gastrointestinal.[1] Given the safety profile of doxycycline and the enrollment of participants receiving ongoing clinical care for HIV or HIV PrEP, study-specific monitoring will be limited to complete blood count and liver function tests at months 3 and 9 to assess for hematologic and hepatic toxicity, which occur rarely with chronic doxycycline use (<1%). Assessment for side effects (eg., pill esophagitis, photosensitivity, new onset headaches), with clinical and laboratory testing if indicated if potential serious adverse events are suspected. Participants will receive standard of care monitoring through their HIV or PrEP. Typical standard of care monitoring for PLHIV includes semi-annual viral load monitoring, renal and liver function tests, and annual CBC. Typical standard of care monitoring for PrEP patients includes quarterly HIV tests and semi-annual or annual renal monitoring.

1.2.2 Rationale for single dose doxycycline as STI PEP.

A concern about a single post-sex dose of doxycycline is the long *in vivo* doubling time of syphilis (approximately 30-33 hours)[31, 32], which is the rationale for treatment of early syphilis with long-acting IM penicillin or oral doxycycline for 14 days. However, the incubation time for syphilis is 10-90 days,[33]and doxycycline PEP administered within 72 hours of exposure may deliver sufficient chemoprophylaxis to abort primary infection. Notably, in IPERGAY, a 73% reduction in syphilis incidence was observed with single dose doxycycline.[1] In

addition, single dose doxycycline PEP has been shown to be effective for Lyme disease[34] and leptospirosis [35, 36] – spirochetal diseases that also have slow doubling times.[35, 37] An alternative approach is doxycycline PrEP (i.e., daily dosing).[22] However, daily doxycycline PrEP may have a higher frequency of intolerability due to side effects, selection of antimicrobial resistance through higher exposure, and greater costs than doxycycline PEP. Importantly, MSM in IPERGAY demonstrated their ability to effectively adhere to peri-coital Truvada PrEP and post-coital doxycycline PEP.

1.2.3 Potential for drug resistance

An important consideration with intermittent doxycycline use for STI prophylaxis is selection of drug resistance, in both target bacterial STI pathogens as well as in microbiota which are potential pathogens or possible reservoirs of antimicrobial resistance, such as *S. aureus* and commensal *Neisseria* species. GC has sequentially developed resistance to antibiotics in multiple classes, including those previously or currently recommended for treatment. Rates of TCN-resistance in GC in the US are 25%-38% in studies of MSM,[11] compared with >50% in Europe.[20] Doxycycline is not currently recommended as treatment for GC. IPERGAY has not reported on TCN resistance in GC and did not address AMR in other bacterial such as *S. aureus* and commensal *Neisseria* species. Doxycycline is commonly used to treat CT and syphilis, and there have been no documented cases of TCN-resistant CT or syphilis. However, TCN resistance from single point mutations has been reported in related organisms such as *Chlamydia suis*[38, 39] and *Brachyspira spp.*[40] in antibiotic-exposed pigs, suggesting that development of TCN resistance could evolve (or be acquired from a resistant organism such as *E. coli* [41]). Azithromycin-resistant *Treponema pallidum* emerged from a single mutation[42] and rapidly became widespread in the US west coast and globally,[43] serving as a cautionary tale.

Repeated antibiotic exposure can foster drug resistance in the normal commensal flora of the body and impact the composition of gut microbiome. The effect of doxycycline use on *S. aureus* is an important consideration, given that ~30% of the population are *S aureus* carriers.[44] AMR is concerning for community-based MRSA infections, for which doxycycline is sometimes used as an alternative to sulfa drugs.[45] Similarly, doxycycline exposure could also drive development of TCN resistance in commensal *Neisseria* species, serving as a reservoir that potentially can transfer resistance to GC. Antibiotics affect the larger microbiome, with decreased diversity of gut flora and shifts in the predominant species, which may fuel a "dysbiotic" environment and may contribute to inflammation and complications such as *C. difficile.*[46-48] In sum, a comprehensive assessment of doxycycline PEP is needed, including public health benefits, safety, tolerability, adherence, and AMR among bacterial STIs and organisms that may can be pathogenic (e.g. *S. aureus* among carriers) or transfer resistance to GC (e.g., from commensal *Neisseria*).

1.2.4 Rationale for evaluation of meningococcal vaccination status

A recent report from New Zealand suggests that vaccination with an outer membrane-based Group B *Neisseria meningitidis* vaccine is associated with a reduced risk of gonorrhea in adolescents and adults aged 15-30 years of age; the estimated effectiveness of the OMV meningococcal vaccine against gonococcal infection to be 33% [NZ OMV is part of the composition of the 4CMenB vaccine, commercially distributed as BEXSERO[49] (GSK). It is not known if other meningococcal vaccines are associated with a similar decrease in GC infections. Meningococcal vaccination (Serogroup A/C/W/Y) is currently recommended by the ACIP for all PLWH and serogroup B vaccination is a consideration for adolescents and young adults[50]. Meningococcal vaccine status will be recorded, with the type of vaccine if known, to evaluate for a potential association of vaccination with decreased GC rates.

1.2.5 Rationale for evaluation of nongonoccal urethritis (NGU) and *Mycoplasma genitalium* (MG)

Mycoplasma genitalium is a frequent cause of urethritis in men .[51] MG can be detected with nucleic acid testing; however, many public health programs do not routinely test for MG and instead treat non-gonoccocal urethritis (NGU) empirically.[52] While azithromycin is superior to doxycycline for treatment of MG, [53, 54] doxycycline does have activity against MG and use of doxycycline PEP may potentially impact the prevalence of MG, the incidence of MG-associated urethritis and the syndrome of NGU. Therefore, urine will be collected every three months to look for the presence of asymptomatic MG as well as at the time of urethral symptoms, to evaluate for MG as the etiology of urethritis.[53, 54] Specimens with MG detected will be stored for future analysis of resistance to tetracycline and other antimicrobials.

1.2.6 Rationale for evaluation of longitudinal weight change

Tetracyclines have been associated with weight gain in mice,[55] as well as in individuals taking tetracycline-based combination therapy for Q fever[56] and for Helicobacter pylori.[57, 58] Therefore, participants in both arms will have weight evaluated at regular intervals to evaluate for possible weight gain

1.2.7 Impact of COVID-19 on study conduct

Study enrollment and the majority of in-person study evaluations were halted in March 2020 due to the COVID-19 pandemic and the hold on clinical research required by the participating institutions. As a result, patients enrolled 3/2020 or earlier were unable to undergo regular ascertainment for the STI endpoint during the following months. Given the need for full endpoint ascertainment and acknowledging potential changes in sexual behavior during the COVID pandemic, participants enrolled 3/2020 and earlier will be offered a full 12 months of follow up at the time of resumption of follow up after COVID restrictions are lifted. Those who decline a 12 month follow up will be discontinued. The team will monitor discontinuation rates in this pre-COVID cohort. If there is a differential discontinuation in the active vs. control arm, this will be discussed with DSMB and the team statistician with consideration of additional enrollments to ensure adequate power for the primaryendpoint

As COVID may continue to impact research activity in the future, the study will allow for electronic consenting as an option, and shipment of doxycycline to participant homes, if necessary and if acceptable to study participants. In-person visits will be preferred when feasible to allow for STI testing, research labs, and pill counts. Home collection of STI testing may be considered, as long as there is sufficient data to support the testing platforms for use in home collection, particularly in terms of syphilis testing.

1.2.8 Early closure by the DSMB and revision to study conduct

Enrollment into the DoxyPEP study was stopped early on 5/13/2022, as a prespecified interim data analysis demonstrated that the study had crossed pre-specified effectiveness thresholds. At the time of interimr review of 554 participants enrolled, a single dose of 200 mg doxycycline taken after condomless sex significantly reduced the acquisition of gonorrhea, chlamydia and syphilis in men who have sex with men (MSM) and transgender women (TGW). The effectiveness of doxycycline PEP was observed and statistically significant both in the cohort of participants living with HIV and the cohort of participants without HIV who were taking PrEP. The strategy of doxyPEP was safe and well-tolerated, with no doxycycline-associated Grade 2 or higher adverse events and no doxycycline-associated serious adverse events.

Enrolled participants will be notified of the study early closure and of the finding of doxycycline effectiveness to reduce STIs. They will also be informed that the impact of doxycycline PEP on antimicrobial resistance in STIs, *S. aureus*, commensal *Neisseria* and the gut microbiome is still under investigation. This protocol modification amends the study to offer doxycycline to enrolled participants assigned to the standard of care arm for the remainder of their 12 month participation. Participants who roll over from the control arm into the active doxycycline PEP arm will undergo the same evaluations as specified for the active arm (e.g., safety labs) per the schedule of events and will continue with quarterly study visits through month 12. At the time of roll over onto active doxycycline PEP, a rectal swab to establish the microbiome baseline prior to starting doxycycline will be collected. Participants who decline to roll over into the active doxycycline arm will have the option of remaining on study through month 12 following the standard of care arm schedule of events. All

participants will review and sign a revised informed consent at the time of their next study visit to consent to continuation in the study and to receive doxycycline, for those in control arm who chose to roll over to active doxyPEP.

The goal of continued follow-up through Month 12 of enrolled participants receiving doxycycline PEP is to obtain additional data on the tolerability and acceptability of doxycycline PEP and the impact of intermittent doxycycline PEP on antimicrobial resistance and the gut microbiome. This information is critical for understanding the risk/benefit profile of doxycycline PEP and to inform guidelines about the doxyPEP strategy which will be considered both by individuals, providers, and public health authorities.

2 STUDY DESIGN

Design: The overarching goal is to assess the effectiveness of doxycycline PEP on incidence of STIs and tetracycline resistance among STIs and commensal bacteria to inform public health policy. Participants will be randomized 2:1 (see Schema), with two-thirds receiving doxycycline PEP and one-third receiving the standard of care control, to maximize data on safety, tolerability, adherence coverage of sexual acts, and resistance data in participants randomized to doxycycline PEP, without negatively impacting power to measure effectiveness. Participants will be counseled about the preliminary effectiveness data from IPERGAY, and the potential for AMR in STIs or other bacteria. Possibility of unreported doxycycline use in the control arm (contamination) will be monitored through retrospective batch testing of doxycycline metabolites in hair, to detect doxycycline use in the prior 3 months. [59]

The trial of doxycycline PEP will be powered to separately assess impact for MSM living with HIV and MSM on PrEP because of potential differences in safety, tolerability, adherence, sexual networks, sexual practices, background AMR, and ultimately, PEP effectiveness. Eligible participants randomized 2:1 to receive PEP will receive open-label doxycycline 200 mg to be taken ideally within 24 hours but no later than 72 hours after condomless sexual contact (oral or anal). 200 mg of doxycycline will be taken at most once per 24 hour period regardless of the number of sexual acts occurring during this time period. the rationale for 2:1 randomization is to maximize data on doxycycline PEP safety, tolerability, acceptability, and resistance, while providing sufficient power for the primary outcome of the effectiveness of doxycycline PEP. Sexual activity will be recorded for both arms of the study (doxycycline PEP and control condition) by the participant using a smartphone application that will be adapted for study use; this will enable comparable assessment of risk in the two arms. PEP pill-taking will also be measured by the app to enable assessment of coverage of sex acts by PEP. Sexual activity and adherence will also be assessed in person at quarterly visits. STI testing will be conducted quarterly from three anatomic sites (pharyngeal, rectal, and urinary) and blood obtained for syphilis testing, following CDC guidelines for serologic diagnosis of syphilis. Participants with a positive STI test will return for STI treatment and for swabs of the affected site for resistance testing; culture based for GC and molecular methods for CT and syphilis. Those with signs and symptoms suggestive of syphilis infection and those with a reactive syphilis serologic test that indicates a new syphilis infection will have swabs of any current active lesion (if present) as well as mucosal swabs from the oropharynx. Swabs from the anterior nares and oropharynx will be obtained at baseline, 6 and 12 months to evaluate tetracycline resistance in *S. aureus* among carriers and oropharyngeal swabs will be obtained at baseline and 12 months to evaluate tetracycline resistance in commensal *Neisseria* species.

Stool samples from 100 participants on the doxycycline PEP arm − 50 MSM and TGW living with HIV and 50 MSM and TGW on PrEP− will be collected at baseline, 6 and 12 months to evaluate effects of intermittent doxycycline on the gut resistome, using FLASH targeted metagenomic sequencing to evaluate for tetracycline and other resistance genes. Rectal swabs will be collected and archived in all participants at baseline, 6, and 12 months for future studies of the impact of doxycycline PEP on the enteric microbiome and resistome.

Study population: This study will enroll 780 participants living with HIV and HIV-negative persons taking PrEP. An approximately equal number of PLWH and HIV uninfected persons taking PrEP will be enrolled. An approximately equal number of participants in each of these cohorts (and in each study arm) will be enrolled in

San Francisco and Seattle.

Current or planned initiation of PrEP use is an eligibility criterion for enrollment, because this population of MSM and TGW has high rates of STIs and is typically seen quarterly for PrEP visits. However, participants may opt to stop PrEP use at any time during the study without affecting their involvement in the study. Any participants without HIV infection who subsequently seroconvert will be managed clinically by the study site according to local practice (appropriate counseling, clinical evaluation, and immediate linkage to clinical and psychosocial services). These participants will also be retained in the study unless they choose to discontinue study participation.

3 OBJECTIVES

3.1 Primary Objectives

3.1.1 Evaluate the effectiveness of doxycycline post-exposure prophylaxis (PEP) to reduce STI infections in two populations: a) MSM/TGW living with HIV and b) MSM/TGW taking HIV PrEP.

3.1.2 Investigate the impact of doxycycline PEP on development of culture-based tetracycline (TCN) resistance in *N. gonorrheae* and among nasopharyngeal carriers of *S. aureus*

3.2 Secondary Objectives

3.2.1 Assess the safety, tolerability, and acceptability of doxycycline PEP

3.2.2 Investigate the impact of doxycycline PEP on detection of culture and molecular markers of tetracycline (TCN) resistance in *C. trachomatis* (CT*), and T. pallidum* (syphilis- molecular markers only).

3.2.3 Measure the proportion of sex acts which are covered by doxycycline PEP

3.2.4 Evaluate the development of phenotypic TCN-resistance among oropharyngeal carriers of commensal *Neisseria* species

3.2.5 Assess the effect of doxycycline PEP on the gut resistome through measuring the abundance of tetracycline resistance genes in stool of a subset of 50 MSM living with HIV and 50 MSM/TGW on PrEP assigned to receive doxycycline PEP as well as rectal swabs from all participants, stored for future evaluation

3.3 Exploratory objectives

3.3.1 Evaluate the diversity of the gut microbiome and quantity and breadth of drug resistance genes in participants on doxycycline PEP and not on PEP

3.3.2 Assess the association between meningococcal vaccination and incident GC infection

3.3.3 Evaluate the incidence of urethritis without identified bacterial etiology and the proportion which is associated with MG in each arm

3.3.4 Assess the rate of detection of *Mycoplasma genitalium* (MG) in urine in each arm and the presence of tetracycline and other antimicrobial resistance in MG in each arm.

3.3.5 Evaluate association of doxycycline use with weight change over time

3.3.6 Evaluate whether doxycycline PEP is associated with PrEP persistence and HIV virologic suppression by comparison of arms and within the doxycycline PEP arm by adherence to doxycycline PEP.

3.3.7 Evaluate the incidence of lymphogranuloma venereum (LGV) in those diagnosed with rectal chlamydia in each arm

4 SELECTION AND ENROLLMENT OF PARTICIPANTS

4.1 Inclusion criteria

4.1.1 Willing and able to give written informed consent

4.1.2 Age ≥ 18 years

4.1.3 Male sex at birth

4.1.4 Previously HIV-diagnosed
OR
HIV-seronegative at the time of last test within the past three months and a current prescription for PrEP (both daily or event-driven PrEP permitted) or plan to start PrEP within 30 days after the enrollment visit

4.1.5 Condomless anal or oral sexual contact with ≥ 1 male sex-at-birth partners in the past 12 months

4.1.6 Diagnosed with GC, CT or syphilis in the past 12 months.
Note: self report of STI is acceptable if documentation not available. If the participant reports an incident STI in the past year at the same clinic where the participant will be enrolled, this diagnosis should be confirmed by chart review prior to enrollment. If the diagnosis from this clinic cannot be confirmed, the participant should not be enrolled. If the STI was reported at a clinical site that is not the study site, and records cannot be obtained, self-report will suffice.

Note: Syphilis diagnosis within the last year refers to primary syphilis, secondary syphilis, and documented early latent syphilis (< 1 year since last syphilis diagnosis or negative test). Known late latent syphilis or latent syphilis of unknown duration would not qualify. Positive syphilis titers which represent serofast status and not active disease do not qualify as a syphilis diagnosis. Clinician judgement regarding qualifying syphilis diagnosis should be sought when the diagnosis of syphilis in the past year is not clear or if there is a question about serofast status vs. active infection.

4.2 Exclusion criteria

4.2.1 Allergy to tetracycline class

4.2.2 Current medications which may impact doxycycline metabolism or that are contraindicated with doxycycline, as per the prescribing information. These include systemic retinoids, barbiturates, carbamazepine, and phenytoin.

4.2.3 Current use of warfarin, as intermittent doxycycline use can lead to an unpredictable impact on INR

4.2.4 Anticipated use of doxycycline during the coming 12 months for non-STI prevention use (e.g., acne treatment).

4.3 Recruitment

Each site has established local recruitment and screening methods that operationalize protocol-specified requirements for eligibility determination in a manner that is tailored to and most efficient for the local study setting and target study population. Each site will use a variety of recruitment approaches, including direct recruitment at clinics, referrals from other providers of PrEP and ART, and use of online and social networking websites and apps. Recruitment materials will educate participants about STI prevalence and incidence in their community and the pilot study that demonstrated efficacy of doxycycline for reducing incidence of STIs among MSM in IPERGAY.

The study will enroll from 4 clinical sites, 2 in Seattle and 2 in San Francisco (Table 1), which have established track records of high quality research in MSM and TGW populations integrated into clinical care settings; annual retention rates in clinical trials conducted in these sites exceed 80%-90%. The sites have large MSM populations that are ethnically and racially diverse.

Table 1. Study Sites

**High volume, comprehensive HIV care clinics with on-site STI testing & treatment.**	• “Ward 86” HIV Clinic, ZSFG, UCSF, San Francsico, CA<br>• Madison (HIV) Clinic, Harborview Medical Center, UW, Seattle, WA
**Municipal STI clinics with integrated on-site HIV PrEP programs.**	• San Francisco City Clinic, San Francisco CA<br>• Public Health – Seattle & King County Sexual Health Clinic, Seattle, WA

4.4 Co-enrollment guidelines.

Participants may be co-enrolled in other research studies, provided that these are a) observational studies or b) evaluation of an FDA approved intervention which is not an antibiotic, is not intended to treat or prevent an STI, and is compatible with coadministration of doxycycline. The study team should be consulted for co-enrollment in studies that do not meet this guidance or if there are questions about eligibility for co-enrollment. For any co-enrolled study, combined blood draws should not exceed current Red Cross phlebotomy guidance.

5 STUDY TREATMENT

5.1 Study product

Generic delayed release doxcycyline hyclate 200 mg capsules (Mayne Pharmaceuticals) will be provided by the study to participants randomized to open-label PEP, and to participants who choose to roll over to active doxycycline PEP following the 5/13/2022 closure of enrollment. Doxycycline DR is bioequivalent to doxycycline hyclate IR.[60, 61] Doxycycline hyclate is widely available and FDA approved for the treatment of multiple infectious conditions. The full prescribing information can be accessed at: https://dailymed.nlm.nih.gov/dailymed/drugInfo.cfm?setid=cd2ab9b8-9619-4199-8a5d-83377b3274d1

5.2 Safety of doxycycline

Doxycycline is an antibiotic that can be used for prolonged periods of several months in the treatment of acne or in the prevention of malaria. The tolerance of repeated-dose of doxycycline in this 12 months study should therefore be acceptable.

The most common side effects of doxycycline are[62, 63]

- Gastrointestinal: nausea, diarrhea, epigastric pain, anorexia, glossitis, enterocolitis, anal and genital candidiasis
- Esophageal disorders: dysphagia, pill esophagitis, and rarely esophageal ulceration. Risk for esophageal irritation can be reduced by taking doxycycline tablets at least one hour before bedtime (to avoid lying down during doxycycline intake) at a meal with a large glass of water (100 ml).
- Skin reactions including maculopapular, erythematous and photosensitivity skin reactions. Due to concern for photosensitivity, individuals taking doxycycline are advised to avoid excessive exposure to sunlight and UV radiation (such as tanning beds).

Other possible but rare adverse effects occurring in < 1% in post-marketing reports include:

- Hypersensitivity reactions: urticarial, anaphylaxis, serum sickness, drug reaction with eosinophilia and systemic symptoms (DRESS)
- Skin toxicity: fixed drug eruption. Stevens-Johnson syndrome, toxic epidermal necrolysis, erythema multiforme, fixed drug eruptions exacerbation of preexisting lupus erythematosus
- Pericarditis
- Hematological disorders: hemolytic anemia, thrombocytopenia, neutropenia, eosinophilia
- Intracranial hypertension (also known as pseudotumor cerebri)

5.3 Doxycycline dispensing and administration.

Doxycycline will be dispensed at each 3-month visit or in shorter intervals per patient preference, with additional dispensation as needed to replace lost medication or provide additional medication when needed (see MOPS for addition dispensing guidance). Capsules should be stored at room temperature (59-86° F) to the extent feasible. Participants will be instructed to take 200 mg of doxycycline within 24 hours after condomless sexual contact, and no later than 72 hours after sex. Condomless sexual contact is defined as receptive anal, insertive anal, vaginal, or oral sex (which includes oral/anal and oral/genital contact) or use of shared sex toys without a condom used for the entire time. For participants reporting multiple sexual encounters, 200 mg of doxycycline will be recommended to be taken every 24 hours until after the last sexual encounter. Participants will be instructed not to exceed 200 mg of doxycycline in a 24 hour period. Study staff will instruct participants on procedures for replacement of lost medication.

Doxycycline may be taken on an empty stomach or with food, and it is advised to take each dose with a large glass of water at least 60 minutes before bed to reduce risk of esophageal irritation. All participants randomized to PEP will be advised to avoid excessive sun exposure and artificial UV light, such as tanning beds, and to consider use of sunscreen if they will be exposed to sun.

5.4 Concomitant medications

Participants randomized to doxycycline PEP should report current and new medications to the study team to ensure no concern for drug interactions with doxycycline. The prescribing information for doxycycline monohydrate should be reviewed to ensure no potential for drug interactions.

5.4.1 Prohibited medications

- Barbiturates
- Carbamazapine
- Phenytoin
- Methoxyflurane
- Systemic retinoids, such as acitretin and isotretinoin

5.4.2 Precautionary Medications

- Antacids containing aluminum, iron, calcium, or magnesium, or bismuth subsalicyclate products (such as Pepto-Bismol) may impair doxycycline absorption and should be separated 2 hours before or after doxycycline
- Oral iron supplements may impact doxycycline absorption. Iron should be separated 2 hours before or after doxycycline

5.5 HIV pre-exposure prophylaxis (PrEP) and HIV antiretroviral therapy (ART)

PrEP and ART will not be provided by the study but will be accessible through local providers following the standard of care. Each of the participating sites has a clinical infrastructure in place to assist individuals with obtaining ART and PrEP, including for those without insurance or whose insurance status changes.

6 CLINICAL AND LABORATORY MONITORING

6.1 Schedule of Events

Month (+2/-10 week window)	*0*	*3*	*6*	*9*	*12 or Termination visit*	*Doxycycline initiation visit for those in control arm*
Informed consent	√					√
Smartphone app instructions	√					
Review of current medications	√	√*	√*	√*	√*	√
Antibiotic & STI history	√	√	√	√	√	
Meningococcal vaccination history	√				√	
Sexual behavior questionnaire	√	√	√	√	√	
Dispense pills/adherence counseling*	√*	√*	√*	√*		√
Doxycycline pill count and adherence recall*		√*	√*	√*	√*	
Doxycycline PEP acceptability survey*			√*		√*	
General symptom assessment	√	√	√	√	√	
Costing questionnaire		√	√	√	√	
Condoms/lubricant	√	√	√	√	√	
Risk reduction counseling	√	√	√	√	√	
GC/CT testing (throat & rectal swab, urine collection)	√	√	√	√	√	
Syphilis testing	√	√	√	√	√	
Rectal swabs stored for future microbiome testing, all participants	√		√		√	√
Rectal swab stored for future MG testing	√	√	√	√	√	
Urine stored for future MG testing (also at time of symptomatic urethritis)	√	√	√	√	√	
Swabs/urine at time of evaluation for STI symptoms or confirmed STIs for resistance testing		*at time of evaluation for STI symptoms or confirmed STI*				
Anterior nares and oropharyngeal swabs for *Staphylococcus aureus* culture	√		√		√	
Oropharyngeal swabs for cultures of Neisseria species	√				√	
Stool sample from first 50 PLWH & first 50 HIV- on PrEP*	√*		√*		√*	
Collection of hair for doxycycline testing			√		√	

HIV testing data from local care (HIV RNA, CD4)	√				√	
CBC and liver function tests*		√*		√*	√**	
Stored serum for future testing (Optional)	√				√	
Reimbursement	√	√	√	√	√	
Weight	√	√	√	√	√	
Qualitative interviews* (subset of participants, optional)		3-12 months				
*Intervention arm only **Standard of care arm only						

6.2 Study visits

Specific study procedures are detailed in Table 6.1. Visits will take place at enrollment and quarterly thereafter, up to 12 months. Interim visits may be scheduled for diagnosis and treatment of STI and collection of additional specimens or for evaluation of possible doxycycline associated adverse events. Participants may also request interim STI testing.

6.2.1 **Enrollment Visit** After confirming eligibility through pre-screening and prior to study procedures, we will obtain written informed consent and collect locating information; the latter will be updated at each study visit. At enrollment, demographic, behavioral, and clinical information will be collected and a release of information obtained to permit access to medical records. STI testing for chlamydia and gonorrhea through nucleic acid amplification testing (NAAT) of clinician or self-collected pharyngeal and rectal swabs and first void urine will be conducted. Serologic testing for syphilis will be conducted. If required STI testing has been conducted within past 30 days, these results may be used for the enrollment evaluation. Participants diagnosed with an STI at baseline will be treated; presence of a baseline STI will not preclude enrollment but STI's present at time of enrollment will not count towards study endpoints. All STIs will be reported to the local health department as required per local standard procedures.

Randomization Within 14 days of enrollment, participants will be randomized in a 2:1 ratio to open-label doxycycline PEP or the standard of care control condition (regular STI counseling, screening and treatment as indicated). The randomization code and resulting allocation list will be generated and maintained by the study statistician. The list will be blocked and stratified by site. While neither participants nor study staff will be blind to each participant's randomization group once assigned, the randomization scheme will utilize varying block sizes in order to protect the blind prior to randomization of each participant.

6.2.2 **Quarterly visits after enrollment**. Quarterly visits will be conducted every 90 days, +2 weeks/ -10 weeks. Participants unable to attend a quarterly visit will be asked to return for an unscheduled visit as soon as feasible. The quarterly evaluations should be completed at the time of this visit. Subsequent visits should occur on the assigned quarterly scheduled to the extent possible.

6.2.3 **Unscheduled interim visits**: Participants will be instructed to contact the study site and return for an unscheduled visit for any of the following a) new diagnosis of STI, b) concern for possible adverse event associated with doxycycline, c) need for additional doxycycline (in participants who were randomized to PEP arm), d) difficulty using the smartphone app, or any other study-related concerns. Reimbursement will not be provided for unscheduled visits. All interim contacts and visits will be documented in participants' study records and on applicable CRFs.

6.2.4 **Termination visit**: In the event that a participant prematurely discontinues study participation, the evaluations for the termination visit should be completed if possible.

6.3 Instructions for evaluations

6.3.1 **Smart phone application**: A smartphone application (app), Blackbook, (developed in conjunction with UCSF researchers) currently in use to track sexual behavior and PrEP use in young MSM has been adapted for use in this study to measure daily sexual risk practices (both study arms) and doxycycline PEP administration (intervention arm only). Study staff will assist with obtaining smartphones through local available resources such as Lifeline Assist, which make cell phones available to qualifying lower income individuals, when needed and feasible (https://www.truconnect.com/lifeline/freephone/signup). Participants will be given unique codes to permit download of the app from an online resource and guidance on how to use the application.

6.3.2 **Review of current medications**: All participants will have current medications reviewed at screening for possible drug interaction with doxycycline. At subsequent quarterly visits, participants randomized to doxycycline PEP will have current medications be reviewed and any new medications will be assessed for possible drug interactions.

6.3.3 **STI symptom screen**. Participants will be asked about current STI symptoms including dysuria, rash, throat pain, rectal pain/discharge, and genital lesions

6.3.4 **Symptom assessment**. At each visit, participants in both study arms will be asked about possible doxycycline side effects, including esophagitis, rash, gastrointestinal symptoms, photosensitivity, headache, and changes in vision (including blurred, double vision, vision loss). Control arm participants will be asked about these symptoms to establish a comparator for non-specific symptoms which can occur in the absence of doxycycline.

6.3.5 **Antibiotic and STI history**: History will be recorded of any non-study provided antibiotics taken in the last 3 months and any STI diagnosis in preceding 3 months, including the diagnosis of urethritis requiring treatment but without detection of gonorrhea or chlamydia. At study entry, STI history for preceding 12 months will be recorded as well as obtained through chart review when possible.

6.3.6 **Meningococcal vaccination history**: At study entry and at month 12, receipt of any prior meningococcal vaccination and the type (if known) will be recorded, using a checklist that will include

- Serogroup A only (includes MenAfriVa, PsA-TT)
- Serogroup B only (includes Trumenba, Bexsero)
- Serogroup C only (includes Meningitec, NeisVac C)
- Quadrivalent A/C/Y/W (includes Menomune, Menveo,Nimirix, Menactra)
- Or other formulation

Type of vaccination may be confirmed by chart review if records are available.

6.3.7 **Sexual behavior questionnaire**: Participants will be asked to complete a sexual behavior questionnaire recording number and type of sexual contacts in the past 3 months at enrollment and between visits

6.3.8 **Pill count and doxycycline adherence assessment**. Participants assigned to doxycycline PEP or who roll over to receive doxycycline in the control arm will be asked to bring their doxycycline bottles in at each visit for a pill count. They will be asked what proportion of sex acts were covered by PEP in the past month and past 3 months, an estimate of overall adherence, and how many total doses of doxycycline were taken in the past 3 months.

Note: any period of 7 or more days in which the participant had condomless sexual activity but did not take doxycycline will be recorded as a doxycycline interruption. This includes interruptions due to lack of doxycycline, forgetting to take doxycycline, taking medication not compatible with doxycycline, or participant decision to temporarily or permanently discontinue study-provided doxycycline.

6.3.9 **Doxycycline PEP acceptability survey**. At months 6 and 12, participants assigned to or now taking doxycycline PEP will be given a brief self-administered questionnaire evaluating acceptability of PEP

6.3.10 **Risk reduction counseling and offer to provide of condoms/lubricant**. At each visit, staff will counsel all participants about STI risk reduction and HIV prevention (if not living with HIV), and the importance of adherence to ART and PrEP, according to local standard of care.

6.3.11 **STI testing**.

GC/CT: First void urine and pharyngeal and rectal swabs will be collected for NAAT testing. Swabs may be self collected or collected by study staff. GC & CT testing should be conducted at least 14 days after treatment to avoid false positive nucleic acid results.

Note: If GC or CT have been conducted within 30 days prior to study visit and these results are available, these results may be used for the quarterly STI assessment. All available STI results should be recorded.

RPR: Blood will be collected for non-treponemal assay (RPR or VDRL) with titer if positive. Treponemal assay results should be recorded if RPR or VDRL are positive. Syphilis testing will be

repeated quarterly after last treatment for syphilis to determine if titers fall or if not, for clinical evaluation of treatment failure or reinfection. Titers must be interpreted in the context of prior test results and current symptoms and exposure history. The need for treatment will be based on the clinical assessment of the site investigator based on the serological data and clinical findings. All syphilis endpoints will be determined by the Endpoint Adjudication committee (see 10.3)

Note: It is preferred to not repeat syphilis testing at a quarterly visit if testing has been done less than 12 weeks before the visit and these tests are available. All available syphilis test results should be recorded.

6.3.12 **Rectal swab for future *M. genitalium* and other testing**.
Rectal swab will be collected and stored for future batched *M.gen* testing, including *M.gen* antibiotic resistance testing, and potential testing for other infectious organisms. Swabs may be self-collected or collected by study staff.

6.3.13 **Urine for *M.genitalium* testing**
At the time of urine collection for GC/CT testing, a sample of this urine collection will be stored for future batched testing for MG. Remnant urine from GC/CT testing may be used or the initial first catch specimen may be divided into two samples, as long as the minimum volume for GC/CT testing is met. See the MOPS for more information. Urine should also be collected for *M.gen* at the time of symptomatic urethritis (see Table 6.4.1)

6.3.14 **Nasal and oropharyngeal swab for *Staphylococcus aureus***: nares and oropharynx will be swabbed and cultured for *S. aureus*, with antibiotic resistance testing if *S. aureus* growth present

6.3.15 **Oropharyngeal swab for commensal Neisseria species**. At baseline and month 12, all participants will undergo oropharyngeal swab for Neisseria species and TCN-resistance testing if Neisseria species are isolated.

6.3.16 **Stool specimen**: Stool samples from the first 50 participants living with HIV and 50 HIV- participants on PrEP who consent to collection will be obtained at baseline, 6 and 12 months for metagenomic resistome studies using 16sRNA amplification of TCN resistance genes. In the event that participants cannot provide a stool sample at the scheduled visit, they will have up to 2 weeks from the enrollment and 12-month visit to return the stool specimens for those visits, and up until two weeks after the 9-month visit to return the stool specimen for the 6-month visit.

6.3.17 **Rectal Swab for microbiome testing**: Rectal swabs will be collected and frozen for future microbiome analysis. These swabs may be self collected or collected by study staff.

6.3.18 **Hair collection**: Hair will be collected from all participants who consent to collection and who meet eligibility for hair collection (occipital hair length > 0.5 cm and absence of bleaching in area to be collected) See manual of procedures for details on hair collection

6.3.19 **HIV testing and RNA data**:
For PLWH: At entry and month 12, the most recent HIV RNA data available from clinical visits will be recorded
For participants on PrEP: At entry, the most recent HIV antibody or viral test results from clinical care will be recorded. If no test results available within the past 3 months, HIV testing should be obtained, consistent with standard of care monitoring for PrEP. At month 12, the most recent HIV antibody or viral load test results from clinical care will be recorded.

6.3.20 **ART and PrEP status**
For PLWH, current ART regimen and status (taking vs. not taking) will be recorded at each visit. At entry and month 12 for PLWH, the most recent HIV RNA and $CD4^{+}$ cell count available from clinical visits will be recorded if available
For participants on PrEP At each visit for those on PrEP, PrEP status will be assessed (taking vs. not taking, and how PrEP is taken- daily vs intermittent). At entry and month 12, the most recent HIV antibody or viral load test results from clinical care will be recorded.

6.3.21 **Hematologic and liver function tests**: Complete blood count (CBC) (white count with differential, hemoglobin, platelets) and liver function testing (AST, ALT, total bilirubin and alkaline phosphatase)

will be drawn at months 3 and 9 in those on doxycycline PEP. Testing done through standard of care within 30 days of the study visit may also be used if available. For those in the standard of care arm, CBC will be done at the month 12 visit. In the event that a CBC cannot be obtained at month 12, an available CBC from any time during study participation may be used.

6.3.22 **Serum for storage** (Optional): At months 0 and 12, 10 mL of serum will be collected for storage for future analysis (non-genetic) in participants who agreed to this. Serum may be collected up to 14 days after the visit if blood cannot be collected at the time of the visit.

6.3.23 **Reimbursement**: Participants will be reimbursed at the enrollment visit, at quarterly visits, and at the time of return for treatment of STIs with accompanying collection of swabs. An additional reimbursement will be provided for participants who consent to the optional stool sample collection at the baseline, 6 month, and 12 month timepoints. No reimbursement will be provided at other unscheduled interim visits such as visits to provide additional doxycycline.

6.3.24 **Roll over from standard of care to active doxycycline:** All participants initially assigned to the standard care arm will be offered the option to receive doxycycline through the study for the remainder of their 12 month follow up period. If a standard of care arm participant elects to initiate doxycycline, they will complete a new informed consent at the doxycycline initiation visit, and will be provided with instructions and counseling on how to use doxycycline PEP. If a control arm participant comes in to initiate doxycycline at a time that falls within the visit window for the next scheduled study visit, all scheduled visit evaluations will be conducted, in addition to a rectal swab for microbiome testing, if not already scheduled. Up to three bottles of doxycycline will be dispensed at the initial visit- a sufficient quantity to ensure enough doxycycline for daily use until the next quarterly visit or the end of study visit. If a control arm participant comes in to initiate doxycycline at a time that is *before* the visit window for the next scheduled study visit, doxycycline will be dispensed and a rectal swab obtained for microbiome evaluation. CBC and LFT evaluations for possible doxycycline toxicity will occur at the month 3 and month 9 study visits per the SOE. If there is a delay in the participant's ability to come in for a doxycycline initiation visit, it is acceptable for doxycycline to be provided through primary care or other provider in advance of visit. Roll over to doxycycline and the date of starting doxycycline will be recorded on a CRF.

6.4 Evaluation at time of presumptive or diagnosed GC, CT and syphilis

6.4.1 Symptoms suggestive of STI for which presumptive STI treatment will be administered.

For those who have symptoms of an STI at a study visit given characteristic clinical presentation (such as chancre, rash characteristic of syphilis, symptomatic urethritis or proctitis) such that the treating clinician will provide empiric STI treatment while awaiting confirmatory testing, standard STI testing should be obtained from urine/pharynx/rectum for GC/CT and blood for syphilis testing. In addition, the following specimens should be obtained for resistance testing from symptomatic anatomic sites based on the clinical presentation as outlined in Table 6.4.1.

NOTE: For specimens collected due to presumptive infection, the resistance testing specimens (swabs/urine collection) should be held until definitive STI testing is available. If STI testing indicates no infection, the specimens may be discarded. Alternatively, the site may send all specimens for storage. Specimens that are not associated with a confirmed STI will be discarded when the data management team confirms absence of STI. If there is uncertainty about whether STI if present, the specimens should be sent to the BioHUB for testing.

NOTE: Testing for evidence of resistance to GC, CT and syphilis should be conducted prior to STI treatment. If collection of specimens before treatment is not possible, CT and syphilis testing can be conducted the same day of treatment after treatment has been provided. Per the SURRG protocol, GC cultures should only be collected before GC treatment.

Table 6.4.1 Specimen collection for resistance testing at time of evaluation for STI and presumptive STI treatment

Presumptive infection		Specimen collection				
	Buccal Swab (Zymo)	Rectal Swab (Zymo)	Urine (Zymo)	Urine (Hologic urine specimen for M.gen)	Swab of chancre/ mucosal lesion (Zymo)	GC culture per local protocol (all anatomic sites of sexual exposure in past 90 days)
Urethritis			√	√		(urethral discharge)
Proctitis		√				√
Syphilis with chancre/mucosal legion					√	
Syphilis diagnosis without chancre/mucosal lesion	√					

6.4.2 Documented STI:

Individuals with positive GC and/or CT NAAT results or with serologic testing indicating a new syphilis infection will be contacted to return for STI treatment as well as swabs of affected site for resistance testing or urine collection, as outlined in Table 6.4.1 Specifically:

- Those with positive GC NAAT results will have
    1) a swab obtained from the involved anatomic site and other sites of sexual contact in past 90 days (to include throat, rectum, urine) if indicated for GC culture-based phenotypic testing according to local GC culture protocol, and
    2) a swab/urine sample obtained from anatomic site of infection only for molecular resistance testing.
- For those with CT infection, a swab or urine specimen will be collected (depending on the site of infection) for molecular resistance testing.
- For those with confirmed rectal or pharyngeal CT, a swab will be collected for chlamydia cell culture. Please see MOPS for specimen handing instructions.
- For those with syphilis serologies indicating new infection and in whom no chancres/mucosal lesions are present, a buccal mucosal swab will be obtained for *T. pallidum* DNA amplification which, if positive, will be used for resistance testing. If suspected syphilitic chancre is present, this should be swabbed for molecular testing

**Documented STI**		**Specimen collection**					
	Buccal Swab (Zymo)	Pharyngeal Swab (Zymo)	Rectal Swab (Zymo)	Rectal or pharyngeal swab (Culture)	Urine Collection (Zymo)	Swab of chancre (Zymo)	GC culture per local protocol (all anatomic sites of sexual exposure in past 90 days
Chlamydia		√*	√*	√*	√*		
Gonorrhea		√*	√*		√*		√
Syphilis with chancre/mucosal legion						√	
Secondary syphilis (rash)	√						

**Specimen sent from site of confirmed infection only*

6.5 Testing for resistance in setting of STI diagnoses on study

6.5.1 Testing for tetracycline resistance among N. gonorroheae

Positive GC cultures for all participants will be sent to either the University of Washington Neisseria Reference laboratory for resistance testing led by our co-investigator, Dr Olusegun Soge at the UW/PHSKC STI program or the SFDPH Public Health Laboratory for testing by Dr. Godfred Masinde. Both laboratories participate in the Strengthening the US Response to Resistant Gonorrhea (SURRG) program. GC isolates collected in Seattle and frozen isolates from San Francisco will be shipped to Dr. Soge on a monthly basis for agar dilution antimicrobial susceptibility to a panel of antimicrobials including tetracycline, as part of the Antimicrobial Resistance Lab Network (ARLN) supported by CDC. In addition, Dr. Soge's laboratory will determine whether tetracycline MICs increases among the subset with repeat GC infections during follow- up.

6.5.2 Molecular testing for tetracycline resistance genes in C. trachomatis, T. pallidum and N. gonorrhoeae.

DNA from positive NAAT tests for *C. trachomatis* and *N. gonorrhoeae* and from buccal or chancre/mucosal lesion swabs from those with newly diagnosed syphilis will be evaluated using established CRISPR/Cas9 techniques to screen against a comprehensive library of antibiotic resistance mutationsin order to establish rapid throughput methodology for the multicenter study. Tetracycline resistance will be assessed using FLASH (Finding Low Abundance Sequences by Hybridization)) targeted sequencing of antimicrobial resistance genes that will then be compared to an established antibiotic resistance database as well as evaluated for known tetracycline mutations that have been documented in the target STIs or closely related organisms[64]. Molecular resistance testing will conducted at the San Francisco Chan-Zuckerberg Biohub under the supervision of collaborating investigator Dr. Langlier, who has extensive expertise in molecular diagnosis of mutations associated with antibiotic resistance. Any TCN-resistance mutations in *C. trachomatis* or *T. pallidum* will be investigated further, as clinically relevant tetracycline resistance has not been reported to date. The results will not be reported for use in clinical care as these methods are experimental and have not been validated for clinical care.

6.6 Treatment of STIs

Local clinic standards will be followed for the treatment of STIs diagnosed by laboratory tests or when identified syndromically. If doxycycline is used for treatment of an STI for an individual in the PEP arm, doxycycyline PEP will be held during treatment and resumed when treatment completed.

6.7 Qualitative interviews

A qualitative interview will be offered to a subset of participants assigned to take doxycycline, approximately 20 per site, during months 3-12, and up to one month after study completion. Qualitative interviews will also be offered to 10-20 providers at each of the sites A separate consent will be provided for these interviews.

6.8 Reenrollment of participants enrolled prior to 3/15/2020

Participants enrolled prior to the COVID pandemic will be re-enrolled starting 7/2020 with the same initial assignment. At time of re-enrollment, these participants will undergo all day 0 evaluations with the exception of the following

- Meningoccal vaccine history
- Medical & STI history
- Stool sample collection from first 50 PLWH and HIV uninfected on PrEP

7 ADVERSE EVENTS (AE) AND STUDY MONITORING

7.1 Adverse Event Collection Requirements

All AEs must be recorded on eCRFs if any of the following criteria have been met:

- All grade 2 and higher hematologic and hepatic laboratory abnormalities that are attributed to doxycycline in the opinion of the site investigator
- All AEs meeting SAE definition
- All grade 3 and 4 adverse events judged to be associated with doxycycline (possibly, probably, or definitely related)

<u>Serious Adverse Events (SAEs)</u>

An SAE is defined as any untoward medical occurrence that:

- Results in death
- Is life-threatening
- Requires inpatient hospitalization or prolongation of existing hospitalization
- Results in persistent or significant disability/incapacity
- Is an important medical event that may not be immediately life-threatening or result in death or

hospitalization but may jeopardize the patient or may require intervention to prevent one of the other outcomes listed in the definition above)

All AEs that are recorded must have their severity graded. To grade AEs, sites should refer to the Division of AIDS Table for Grading the Severity of Adult and Pediatric Adverse Events (DAIDS AE Grading Table), corrected Version 2.1, July 2017, which can be found on the DAIDS RSC website at https://rsc.tech-res.com/docs/default-source/safety/division-of-aids-(daids)-table-for-grading-the-severity-of-adult-and-pediatric-adverse-events-corrected-v-2-1.pdf

7.2 AE and SAE attribution to doxycycline

In those randomized to receive doxycycline PEP, all AEs and SAEs should have attribution recorded as doxycycline-related or not doxycycline-related, in the judgment of the site investigator.

7.3 Study Monitoring

7.3.1 Study Team Monitoring

The study team will monitor the conduct of the study through monthly summary reports of arms of accrual, and baseline characteristics and quarterly reports of data pooled over treatment arms of data completeness, specimen collection, and AEs. The study will review individual participant-level safety data frequently to assess the relation of all reported AEs to study treatment. On a monthly basis, the study team will review by- arm summaries of premature study discontinuations and premature study treatment discontinuations (and reasons) and AEs.

7.3.2 Independent Monitor

Study conduct will be monitored by an independent monitor. Monitors will visit participating clinical research sites to review the individual participant records, including consent forms, eCRFs, supporting data, laboratory specimen records, and endpoints through laboratory and medical records (physicians' progress notes, nurses' notes, individuals' hospital charts), to ensure protection of study participants, compliance with the protocol, and accuracy and completeness of records. The monitors also will inspect sites' regulatory files to ensure that regulatory requirements are being followed and sites' pharmacies to review product storage and management.

7.3.3 Data safety and monitoring board (DSMB)

An independent data safety and monitoring board (DSMB) will be convened for this study with expertise in STIs, PrEP, and HIV care and a biostatistician. The purpose of the DSMB is to monitor the study for operational futility, social harms, and efficacy. The DSMB will evaluate the progress of the project, including periodic assessments of accrual, retention, safety, performance and variation of the project sites, and other factors that can affect project implementation. The DSMB will review the study after 1/2 of follow- up time with pre-specified stopping rules in terms of the efficacy of doxycycline PEP in reducing the incidence of gonorrhea, chlamydia, and syphilis overall, and the ability of the study to meet its objectives. The DSMB will review STI endpoints which have been adjudicated by the STI Endpoints Committee. The DMSB will consider factors external to the project when relevant information becomes available, such as policy changes or scientific developments that may have an impact on project implementation, safety, and integration of STI PEP in the STI and HIV care clinics.

The DSMB will conduct reviews every six month and convene by teleconference. Open reports containing accrual and retention rates, participant characteristics, serious adverse events, and social, will be sent to the protocol team and DSMB members the week prior to the DSMB meeting. Only the DSMB members and the unblinded biostatistician will receive password-protected closed reports of STI endpoints by randomization arm.

8 CLINICAL MANAGEMENT ISSUES

8.1 Toxicity

Only toxicities related to study medications provided through the study will be considered in the toxicity management section.

Grade 1 or 2

Participants who develop Grade 1 or 2 toxicity (per DAIDS toxicity table) felt to be related to doxycycyline may continue study treatment at the discretion of the site investigator with close follow-up. If a participant chooses to discontinue study treatment, the site should notify the study protocol team within 7 days.These participants will remain on study, off study treatment and have all evaluations performed per the SOE.

Grade 3

- Participants who develop a Grade 3 toxicity thought by the site investigator to be related to study drug should have doxycycline PEP withheld and the site should consult with the core protocol team. The participant should be reevaluated weekly until the AE returns to Grade ≤2, at which time study drug may be reintroduced at the discretion of the site investigator in consultation with the protocol team.
- Participants experiencing Grade 3 toxicity requiring permanent discontinuation of doxycycline PEP should be followed weekly until resolution of the toxicity. Participants will have premature study treatment discontinuation evaluations performed as noted on the SOE. These participants will remain on study, off study treatment and have all evaluations performed per the SOE.

Grade 4

- Participants with Grade 4 asymptomatic laboratory abnormalities may continue doxycycline PEP if the site investigator has compelling evidence that the toxicity is NOT related to study drug.
- Participants who develop a Grade 4 symptomatic toxicity will have doxycycline PEP permanently discontinued and the site should notify study team within 72 hours.
- Participants experiencing Grade 4 toxicity requiring permanent discontinuation of doxycycline PEP should be followed weekly until resolution of the AE or return to baseline. These participants will remain on study, off study treatment and have all evaluations performed per the SOE.
-

8.2 Specific Management of Toxicities Related to Study-Provided Drugs

Possible intracranial hypertension (IH)

Participants taking doxycycline who report new or worsening headaches, visual changes, or vision loss should have doxycycline temporarily discontinued and evaluated for IH if these symptoms persist, including a fundoscopic exam to look for papilledema. If IH is ruled-out or an alternate etiology identified, doxycycline may be restarted, at the discretion of the site investigator.

Skin erythema

Increased photosensitivity is a known possible side effect of doxycycline. Doxycycline may be discontinued if skin erythema develops at the discretion of the site investigator and may reinstituted when resolved at the discretion of the site investigation. Any serious skin reaction judged by the local investigator to be related to doxycycline should lead to permanent discontinuation of doxycycline .

Fixed drug eruption

Suspected fixed drug eruption should be evaluated for possible etiologies – if a doxycycline fixed drug eruption is suspected, doxycycline should be stopped.

Allergic reactions

Doxycycline should be discontinued permanently if a serious allergic reaction is suspected. These participants will remain on study, off study treatment and have all evaluations performed per the SOE.

9 CRITERIA FOR DISCONTINUATION

9.1 Premature Study Treatment Discontinuation

- Requirement for prohibited concomitant medications or other contraindication to doxycycline
- Occurrence of an adverse event requiring discontinuation of doxycycline
- Request by participant to terminate study treatment
- Clinical reasons believed life threatening by the physician, even if not addressed in the toxicity section of the protocol
- Requirement for chronic tetracycline use (>14 days)

Participants who stop doxycycline PEP should be continued on study, off doxycycline PEP with continued evaluations as per the SOE. The reason for doxycycline discontinuation should be recorded.

9.2 Premature Study Discontinuation

- Request by the participant to withdraw
- Request of the primary care provider if she or he thinks the study is no longer in the best interest of the participant
- Participant judged by the investigator to be at significant risk of failing to comply with the provisions of the protocol as to cause harm to self or seriously interfere with the validity of the study results
- At the discretion of the IRB/EC, NIAID, Office for Human Research Protections (OHRP), other government agencies as part of their duties, investigator, or industry supporter

10 STATISTICAL CONSIDERATIONS

10.1 Outcome measures

10.1.1 Primary outcome:

Combined incidence of GC, CT, or early syphilis infection by laboratory- based diagnosis (e.g., positive GC or CT based on NAAT, syphilis based on four-fold increase in non- treponemal titers or positive darkfield on exudate from a lesion). Incident STI diagnoses will be ascertained through the study through periodic testing, interim visits for STI symptoms, as well as STI surveillance from the San Francisco and King County Departments of Health and by medical records provided for participants who receive their care from other clinical sites.

10.1.2 Secondary outcomes:

- Safety of doxycycline PEP, measured by evaluation of all Grade 3 and 4 AEs attributed to study medication.
- Tolerability, measured using a brief quarterly questionnaire administered to both intervention and control groups evaluating side effects commonly associated with doxycycline as well as recording any discontinuations of doxycycline by participants and their reasons.
- Acceptability, assessed through a brief questionnaire for all PEP recipients administered at month 6 and 12 and in-depth structured qualitative interviews for a subset, conducted within 6 months of month 12 visit. A separate consent will be provided for the in-depth qualitative interview at the time of this interview.
- Doxycycline coverage of sexual contacts in the prior 3 months, ascertained by sexual behavior and doxycycline

use from the web-based app, augmented by quarterly adherence questionnaires and pill counts.

- Longitudinal exposure to doxcycyline will be assessed using hair collection, with a semiquantitive evaluation in the control arm (doxycycline present vs. absent) and in the PEP arm (doxycycline concentration high vs. low).
- Adherence to HIV medications as reflected in viral suppression among participants living with HIV, assessed by chart review of HIV RNA levels conducted as standard clinical care.
- Discontinuation of PrEP, based on self-report
- Residual hair samples of participants without HIV infection on PrEP will be archived for future tenofovir testing to measure adherence to Truvada PrEP.

10.2 Statistical power and analysis

Primary endpoint: We selected a sample size for each cohort (MSM/TGW living with HIV and MSM/TGW on PrEP) to achieve 80% power with 0.05 two-sided Type 1 error, for the endpoint of any lab-detected incident early syphilis, GC and/or CT infection, based on quarterly assessments for each person, 10% annual loss to follow-up and an intra-class correlation of 0.2, reflecting the high prevalence of STI re-infection that has been observed in these populations. With these assumptions, a cohort of approximately 390 of MSM/TGW living with HIV and 390 of MSM/TGW on PrEP (130 in the control and 260 in the intervention for each cohort) will provide 80% power to detect a decrease in quarterly STI prevalence from 10% to 5%, which corresponds to an annual reduction in combined STI incidence from 34% to 19% (Table 10.2.1). With allowance for 10% lost to follow-up, the sample size for each cohort will be approximately 390 participants, for a total sample size of 780. Based on 2017 STI incidence data from San Francisco and Seattle PrEP cohorts for both GC and CT, we expect in the control arm in each quarter approximately 5% of participants to have GC, 5% CT and 2% early syphilis, acknowledging some will be diagnosed with more than one infection. Based on the higher susceptibility of GC to TCN in the US vs France (75% vs < 50%[11, 20]) and the 70% reduction of CT and syphilis with PEP in IPERGAY,[1] we anticipate effectiveness of doxycycline PEP to be 35% for GC, 65% for CT and 65% for syphilis. Given these assumptions, with a 2:1 randomization we expect to see 61 vs 47 GC infections, 47 vs 33 CT infections and 19 vs 13 syphilis infections in the doxycycline PEP and control arms respectively, some of which will occur in the same individuals. Sample sizes were computed using nQuery for repeated measures for two proportions. The analysis will compare time-averaged proportion of infections in each quarter between arms, using repeated measure methods.

Table 10.2.1: Power Table for N = 390; 2:1 randomization doxycycline PEP: SOC. Sample size assumes 370 fully evaluable participants.

Control arm quarterly prevalence	Intervention arm quarterly prevalence	Power to detect 50% reduction		Power to detect 65% reduction	
		ICC = 0.2	ICC = 0.1	ICC = 0.2	ICC = 0.1
10%	5%	80%	87%	96%	99%
7.5%	3.75%	68%	76%	90%	95%
5%	2.5%	51%	59%	77%	85%
3%	1.5%	35%	41%	57%	65%
2%	1%	26%	30%	43%	50%

Within the total enrollment of 780, we will not require the enrollment of HIV-infected and uninfected participants to be equal. Anticipating the possibility of lower enrollment but possibility of equivalent or higher STI rates in the HIV-infected cohort, Table 10.2.2 assesses the power characteristics of the study with a cohort that contributes from 30% to 50% of the total enrolled, with quarterly events rates of 10% and 12%. With a higher event rate or a lower intraclass correlation, the smaller cohort retains reasonable power to detect a 50% reduction.

Table 10.2.2: **Power characteristics for reduced sample size in a cohort, assuming event rate of 10% and 12% STIs occurring each quarter in SOC arm**

Total Evaluable sample size in cohort	ICC	Doxy: SOC	Power to detect 50% reduction (10% in SOC)	Power to detect 50% reduction (12% in SOC)
370 (50% of total)	0.1	246:123	87%	92%
370	0.2	246:123	80%	86%
296 (40% of total)	0.1	196:98	79%	86%
296	0.2	196:98	71%	78%
259 (35% of total)	0.1	174:87	74%	82%
259	0.2	174:87	66%	74%
222 (30% of total)	0.1	148:74	68%	76%
222	0.2	148:74	60%	67%

Secondary endpoints include evaluation of PEP effectiveness on incidence of each specific STI and on time to first STI, as well as by site of STI infection (oral, rectal, urethral). Impact of risk practices and sexual activity on STI incidence will be compared between arms. Survey data will be analyzed to understand factors impacting acceptability of doxycycline PEP, including demographic factors, socioeconomic status, substance use, history of STIs, HIV infection status, and experience with PEP. The qualitative interviews will be coded using inductive (i.e., codes arise from careful reading of the text) and deductive (i.e., codes arise *a priori* from topics covered by the interview guide) approaches, which when employed together have proven useful in health services research. Interviews will be coded by at least two research team members who will practice on a selection of transcripts until >90% agreement is reached. Dedoose, a web-based program, will be used to facilitate indexing, coding, and searching. The qualitative team will meet regularly to group codes into categories and develop major and minor themes.

For resistance we will assess the proportion with tetracycline resistance in visits with *N. gonorrheae* and *S. aureus*. Exploratory endpoints include incidence of tetracycline resistance in *C. trachomatis* and *T. pallidum* using molecular techniques (e.g., CRISPR and CAS-9), for which the molecular technologies are new and the clinical significance of resistance mutations is less clear. Stool samples from 50 participants living with HIV and 50 participants on PrEP will be obtained at baseline, 6 and 12 months for metagenomic evaluation targeting TCN genes. Overall TCN resistance gene abundance (expressed as dpM/patient) and the number of distinct TCN resistance genes present per participant will be assessed at baseline and evaluated for change over time during doxycycline use. Rectal swabs will be collected and stored for future evaluation of shifts in the fecal microbiome, decrease in flora diversity, and quantification of TCN-resistant abundance and diversity

10.3 Endpoint adjudication

All STI endpoints will be reviewed by a blinded, independent Endpoint Adjudication Committee, following CDC guidelines for the diagnosis of STIs.[52] Incidence of individual STIs and tetracycline resistance will also be evaluated.

10.4 Post COVID follow-up

The team will monitor discontinuation rates in the participants enrolled prior to the COVID epidemic (3/15/2020). If there is a differential discontinuation in the active vs. control arm, this will be discussed with DSMB and the team statistician with consideration of additional enrollments to ensure adequate power for the primary endpoint.

10.5 Analyses after 5/13/2022 closure to enrollment

Analyses after 5/13/2022 closure to enrollment and doxycycline provision to control will include the following:

STI incidence:

- Comparison of STI incidence per quarter in control arm before and after doxycycline initiation
- Comparison of STI incidence per quarter in all participants receiving doxycycline (including those in control arm after rolled over to receive doxycycline) compared to incidence on control arm prior to doxycycline roll over

Antimicrobial resistance

- Evaluation of tetracycline resistance in *Staphylococcus aureus*, commensal *Neisseria* in comparison to the control arm and to individual baseline
- Evaluation of genotypic or phenotypic tetracycline resistance in bacterial STIs in all participants who receive doxycycline compared to control arm participants, prior to receipt of doxycycline.
- Evaluation of changes in the gut microbiome and total gut burden of tetracycline resistance before and after taking doxycycline PEP

Adherence

- Evaluation of doxycycline coverage of qualifying sex before and after study closure 5/13/2022

Acceptability

- Proportion of control arm participants who opt to take study provided doxycycline. Control arm participants who terminate study participation will have the reason for termination recorded to determine if stopping to receive doxycycline outside of the study.

11 PARTICIPANTS

11.1 Institutional Review Board (IRB) Review

The study protocol, site-specific informed consent forms, participant education and recruitment materials, and other requested documents—including any subsequent modifications—will be reviewed and approved by the UCSF IRB, as the single IRB of record responsible for oversight of research conducted at the study sites. Subsequent to initial review and approval, the UCSF IRB will review the study at least annually.

11.2 Informed Consent

A signed consent form will be obtained from the participant.The consent form will describe the purpose of the study, the procedures to be followed, and the risks and benefits of participation. A copy of the consent form will be given to the participant and this fact will be documented in the participant's record. Electronic consenting will be an option to reduce face to face exposure during the COVID epidemic.

11.3 Study records

Each study site will establish a standard operating procedure for confidentiality protection. Each site will ensure that study records including administrative documentation and regulatory documentation as well as documentation related to each participant enrolled in the study, including informed consent forms, locator forms, case report forms, notations of all contacts with the participant, and all other source documents are stored in a secure manner.

11.4 Confidentiality

Participants' study information will not be released without their written permission, except as necessary for oversight by:

- The Protocol co-Chairs or designees
- Study funders
- University of California, San Francisco IRB
- University of Washington IRB

All laboratory specimens, evaluation forms, reports, and other records that leave the site will be identified by coded number only to maintain participant confidentiality. The exceptions are STI testing results which are subject to local and state reporting which is names-based. Local public health may contact participants diagnosed with STIs for the purpose of surveillance and partner notification. Participants will be informed prior to STI testing that results are reportable and may lead to contact by local public health if results are positive for infection. In addition, GC culture specimens will be evaluated for drug resistance as part of an existing GC program evaluating clinical isolates and may contain patient identifiers such as MRN, name or date of visit, according to local laboratory requirements and practice.

All records will be kept locked. All computer entry and networking programs will be done with coded numbers only. Clinical information will not be released without written permission of the participant, except as necessary for monitoring by the IRB/EC, NIAID, OHRP, other local, US, and international regulatory entities as part of their duties, or the industry supporters or designees.

12 BIOHAZARD CONTAINMENT

As the transmission of HIV and other blood-borne pathogens can occur through contact with contaminated needles, blood, and blood products, appropriate blood and secretion precautions will be employed by all personnel in the drawing of blood and shipping and handling of all specimens for this study, as currently recommended by the CDC and the National Institutes of Health.

All dangerous goods and materials, including diagnostic specimens and infectious substances, must be transported using packaging mandated by CFR 42 Part 72. Please refer to instructions detailed in the International Air Transport Association (IATA) Dangerous Goods Regulations.

REFERENCES

1. Molina, J.M., I. Charreau, C. Chidiac, G. Pialoux, E. Cua, C. Delaugerre, C. Capitant, D. Rojas-Castro, J. Fonsart, B. Bercot, C. Bebear, L. Cotte, O. Robineau, F. Raffi, P. Charbonneau, A. Aslan, J. Chas, L. Niedbalski, B. Spire, L. Sagaon-Teyssier, D. Carette, S.L. Mestre, V. Dore, and L. Meyer, *Post-exposure prophylaxis with doxycycline to prevent sexually transmitted infections in men who have sex with men: an open-label randomised substudy of the ANRS IPERGAY trial.* Lancet Infect Dis, 2017.

2. CDC, *Sexually Transmitted Disease Surveillance 2016*. 2017.

3. Volk, J.E., J.L. Marcus, T. Phengrasamy, D. Blechinger, D.P. Nguyen, S. Follansbee, and C.B. Hare, *No New HIV Infections With Increasing Use of HIV Preexposure Prophylaxis in a Clinical Practice Setting.* Clin Infect Dis, 2015. **61**(10): p. 1601-3.

4. Montano, M.A., J.C. Dombroski, L.A. Barbee, M.R. Golden, and C.M. Khosropour. *Changes in sexual behavior and STI diagnoses among MSM using PrEP in Seattle, WA (Abstract #979)*. in *Conference on Retroviruses and Opportunistic Infections (CROI)*. 2018. Boston, MA.

5. Oliver, S.E., M. Aubin, L. Atwell, J. Matthias, A. Cope, V. Mobley, A. Goode, S. Minnerly, J. Stoltey, H.M. Bauer, R.R. Hennessy, D. DiOrio, R.N. Fanfair, T.A. Peterman, and L. Markowitz, *Ocular Syphilis - Eight Jurisdictions, United States, 2014-2015.* MMWR Morb Mortal Wkly Rep, 2016. **65**(43): p. 1185-1188.

6. Bowen, V., J. Su, E. Torrone, S. Kidd, and H. Weinstock, *Increase in incidence of congenital syphilis - United States, 2012-2014.* MMWR Morb Mortal Wkly Rep, 2015. **64**(44): p. 1241-5.

7. Cunningham, S.D., G. Olthoff, P. Burnett, A.M. Rompalo, and J.M. Ellen, *Evidence of heterosexual bridging among syphilis—positive men who have sex with men.* Sexually Transmitted Infections, 2006. **82**(6): p. 444-445.

8. Wi, T., M.M. Lahra, F. Ndowa, M. Bala, J.-A.R. Dillon, P. Ramon-Pardo, S.R. Eremin, G. Bolan, and M. Unemo, *Antimicrobial resistance in Neisseria gonorrhoeae: Global surveillance and a call for international collaborative action.* PLOS Medicine, 2017. **14**(7): p. e1002344.

9. Gianecini, R., C. Oviedo, G. Stafforini, and P. Galarza, *Neisseria gonorrhoeae Resistant to Ceftriaxone and Cefixime, Argentina.* Emerging Infectious Diseases, 2016. **22**(6): p. 1139-1141.

10. Camara, J., J. Serra, J. Ayats, T. Bastida, D. Carnicer-Pont, A. Andreu, and C. Ardanuy, *Molecular characterization of two high-level ceftriaxone-resistant Neisseria gonorrhoeae isolates detected in Catalonia, Spain.* J Antimicrob Chemother, 2012. **67**(8): p. 1858-60.

11. Kirkcaldy, R.D., A. Harvey, J.R. Papp, C. Del Rio, O.O. Soge, K.K. Holmes, E.W. Hook, 3rd, G. Kubin, S. Riedel, J. Zenilman, K. Pettus, T. Sanders, S. Sharpe, and E. Torrone, *Neisseria gonorrhoeae Antimicrobial Susceptibility Surveillance - The Gonococcal Isolate Surveillance Project, 27 Sites, United States, 2014.* MMWR Surveill Summ, 2016. **65**(7): p. 1-19.

12. BBC. *'World's worst' super-gonorrhoea man cured*. 2018 4/26/2018]; Available from: http://www.bbc.com/news/health-43840505.

13. Lahra, M.M., N. Ryder, and D.M. Whiley, *A new multidrug-resistant strain of Neisseria gonorrhoeae in Australia.* N Engl J Med, 2014. **371**(19): p. 1850-1.

14. Cohen, M.S., I.F. Hoffman, R.A. Royce, P. Kazembe, J.R. Dyer, C.C. Daly, D. Zimba, P.L. Vernazza, M. Maida, S.A. Fiscus, and J.J. Eron, Jr., *Reduction of concentration of HIV-1 in semen after treatment of urethritis: implications for prevention of sexual transmission of HIV-1. AIDSCAP Malawi Research Group.* Lancet, 1997. **349**(9069): p. 1868-73.

15. Schacker, T., A.J. Ryncarz, J. Goddard, K. Diem, M. Shaughnessy, and L. Corey, *Frequent recovery of HIV-1 from genital herpes simplex virus lesions in HIV-1-infected men.* JAMA, 1998. **280**(1): p. 61-6.

16. CDC. *Understanding the HIV Care Continuum*. 2017; Available from: https://www.cdc.gov/hiv/pdf/library/factsheets/cdc-hiv-care-continuum.pdf.

17. The Lancet, H., *U=U taking off in 2017.* Lancet HIV, 2017. **4**(11): p. e475.

18. Harrison, W.O., R.R. Hooper, P.J. Wiesner, A.F. Campbell, W.W. Karney, G.H. Reynolds, O.G. Jones, and K.K. Holmes, *A trial of minocycline given after exposure to prevent gonorrhea.* N Engl J Med, 1979. **300**(19): p. 1074-8.

19. Steen, R., M. Chersich, A. Gerbase, G. Neilsen, A. Wendland, F. Ndowa, E.A. Akl, Y.R. Lo, and S.J. de Vlas, *Periodic presumptive treatment of curable sexually transmitted infections among sex workers: a systematic review.* Aids, 2012. **26**(4): p. 437-45.

20. La Ruche, G., A. Goubard, B. Bercot, E. Cambau, C. Semaille, and P. Sednaoui, *Gonococcal infections and emergence of gonococcal decreased susceptibility to cephalosporins in France, 2001 to 2012.* Euro Surveill, 2014. **19**(34).

21. Molina, J.M., I. Charreau, C. Chidiac, G. Pialoux, E. Cua, C. Delaugerre, C. Capitant, D. Rojas-Castro, J. Fonsart, B. Bercot, C. Bebear, L. Cotte, O. Robineau, F. Raffi, P. Charbonneau, A. Aslan, J. Chas, L. Niedbalski, B. Spire, L. Sagaon-Teyssier, D. Carette, S.L. Mestre, V. Dore, and L. Meyer. *Supplement to: Molina JM, Charreau I, Chidiac C, et al. Post-exposure prophylaxis with doxycycline to prevent sexually transmitted infections in men who have sex with men: an open-label randomised substudy of the ANRS IPERGAY trial. Lancet Infect Dis 2017*. 2017; Available from: https://www.sciencedirect.com/science/article/pii/S1473309917307259?via%3Dihub#sec1.

22. Bolan, R.K., M.R. Beymer, R.E. Weiss, R.P. Flynn, A.A. Leibowitz, and J.D. Klausner, *Doxycycline prophylaxis to reduce incident syphilis among HIV-infected men who have sex with men who continue to engage in high-risk sex: a randomized, controlled pilot study.* Sex Transm Dis, 2015. **42**(2): p. 98-103.

23. Gaillard, T., M. Madamet, and B. Pradines, *Tetracyclines in malaria.* Malar J, 2015. **14**: p. 445.

24. van Zuuren, E.J. and Z. Fedorowicz, *Interventions for rosacea: abridged updated Cochrane systematic review including GRADE assessments.* Br J Dermatol, 2015. **173**(3): p. 651-62.

25. Bienenfeld, A., A.R. Nagler, and S.J. Orlow, *Oral Antibacterial Therapy for Acne Vulgaris: An Evidence-Based Review.* Am J Clin Dermatol, 2017. **18**(4): p. 469-490.

26. Sloan, B. and N. Scheinfeld, *The use and safety of doxycycline hyclate and other second-generation tetracyclines.* Expert Opin Drug Saf, 2008. **7**(5): p. 571-7.

27. Turner, R.B., C.B. Smith, J.L. Martello, and D. Slain, *Role of doxycycline in Clostridium difficile infection acquisition.* Ann Pharmacother, 2014. **48**(6): p. 772-6.

28. Tariq, R., J. Cho, S. Kapoor, R. Orenstein, S. Singh, D.S. Pardi, and S. Khanna, *Low Risk of Primary Clostridium difficile Infection With Tetracyclines: A Systematic Review and Metaanalysis.* Clin Infect Dis, 2018. **66**(4): p. 514-522.

29. Doernberg, S.B., L.G. Winston, D.H. Deck, and H.F. Chambers, *Does doxycycline protect against development of Clostridium difficile infection?* Clin Infect Dis, 2012. **55**(5): p. 615-20.

30. Khanna, S., *Do tetracyclines have the potential to reduce the risk of Clostridium difficile infection?* Expert Review of Anti-infective Therapy, 2018: p. 1-3.

31. Magnuson, H.J., H. Eagle, and R. Fleischman, *The minimal infectious inoculum of Spirochaeta pallida (Nichols strain) and a consideration of its rate of multiplication in vivo.* Am J Syph Gonorrhea Vener Dis, 1948. **32**(1): p. 1-18.

32. Cumberland, M.C. and T.B. Turner, *The rate of multiplication of Treponema pallidum in normal and immune rabbits.* Am J Syph Gonorrhea Vener Dis, 1949. **33**(3): p. 201-12.

33. Lafond, R.E. and S.A. Lukehart, *Biological basis for syphilis.* Clin Microbiol Rev, 2006. **19**(1): p. 29-49.

34. Nadelman, R.B., J. Nowakowski, D. Fish, R.C. Falco, K. Freeman, D. McKenna, P. Welch, R. Marcus, M.E. Aguero-Rosenfeld, D.T. Dennis, and G.P. Wormser, *Prophylaxis with single-dose doxycycline for the prevention of Lyme disease after an Ixodes scapularis tick bite.* N Engl J Med, 2001. **345**(2): p. 79-84.

35. Levett, P.N., *Leptospirosis.* Clin Microbiol Rev, 2001. **14**(2): p. 296-326.

36. Schneider, M.C., J. Velasco-Hernandez, K.D. Min, D.G. Leonel, D. Baca-Carrasco, M.E. Gompper, R. Hartskeerl, and C. Munoz-Zanzi, *The Use of Chemoprophylaxis after Floods to Reduce the Occurrence and Impact of Leptospirosis Outbreaks.* Int J Environ Res Public Health, 2017. **14**(6).

37. Burkot, T.R., J. Piesman, and R.A. Wirtz, *Quantitation of the Borrelia burgdorferi outer surface protein A in Ixodes scapularis: fluctuations during the tick life cycle, doubling times, and loss while feeding.* J Infect Dis, 1994. **170**(4): p. 883-9.

38. Wanninger, S., M. Donati, A. Di Francesco, M. Hässig, K. Hoffmann, H.M.B. Seth-Smith, H. Marti, and N. Borel, *Selective Pressure Promotes Tetracycline Resistance of Chlamydia Suis in Fattening Pigs.* PLOS ONE, 2016. **11**(11): p. e0166917.

39. Dugan, J., D.D. Rockey, L. Jones, and A.A. Andersen, *Tetracycline resistance in Chlamydia suis mediated by genomic islands inserted into the chlamydial inv-like gene.* Antimicrob Agents Chemother, 2004. **48**(10): p. 3989-95.

40. Mirajkar, N.S., P.R. Davies, and C.J. Gebhart, *Antimicrobial Susceptibility Patterns of Brachyspira Species Isolated from Swine Herds in the United States.* J Clin Microbiol, 2016. **54**(8): p. 2109-19.

41. Centurion-Lara, A., C. Castro, W.C. van Voorhis, and S.A. Lukehart, *Two 16S-23S ribosomal DNA intergenic regions in different Treponema pallidum subspecies contain tRNA genes.* FEMS Microbiol Lett, 1996. **143**(2-3): p. 235-40.

42. Mitchell, S.J., J. Engelman, C.K. Kent, S.A. Lukehart, C. Godornes, and J.D. Klausner, *Azithromycin-resistant syphilis infection: San Francisco, California, 2000-2004.* Clin Infect Dis, 2006. **42**(3): p. 337-45.

43. Stamm, L.V., *Global challenge of antibiotic-resistant Treponema pallidum.* Antimicrob Agents Chemother, 2010. **54**(2): p. 583-9.

44. Lucet, J.-C. and B. Regnier, *Screening and Decolonization: Does Methicillin-Susceptible Staphylococcus aureus Hold Lessons for Methicillin-Resistant S. aureus?* Clinical Infectious Diseases, 2010. **51**(5): p. 585-590.

45. Stevens, D.L., A.L. Bisno, H.F. Chambers, E.P. Dellinger, E.J. Goldstein, S.L. Gorbach, J.V. Hirschmann, S.L. Kaplan, J.G. Montoya, J.C. Wade, and A. Infectious Diseases Society of, *Practice guidelines for the diagnosis and management of skin and soft tissue infections: 2014 update by the Infectious Diseases Society of America.* Clin Infect Dis, 2014. **59**(2): p. e10-52.

46. Jakobsson, H.E., C. Jernberg, A.F. Andersson, M. Sjölund-Karlsson, J.K. Jansson, and L. Engstrand, *Short-Term Antibiotic Treatment Has Differing Long-Term Impacts on the Human Throat and Gut Microbiome.* PLOS ONE, 2010. **5**(3): p. e9836.

47. Shanahan, F., *The colonic microbiota in health and disease.* Curr Opin Gastroenterol, 2013. **29**(1): p. 49-54.

48. Blaser, M.J., *The Past and Future Biology of the Human Microbiome in an Age of Extinctions.* Cell, 2018. **172**(6): p. 1173-1177.

49. Petousis-Harris, H., J. Paynter, J. Morgan, P. Saxton, B. McArdle, F. Goodyear-Smith, and S. Black, *Effectiveness of a group B outer membrane vesicle meningococcal vaccine against gonorrhoea in New Zealand: a retrospective case-control study.* Lancet, 2017. **390**(10102): p. 1603-1610.

50. ACIP. *Recommended Immunization Schedule for Adults Aged 19 Years or Older, United States, 2018*. 2018; Available from: https://www.cdc.gov/vaccines/schedules/hcp/imz/adult.html#f10.

51. Taylor-Robinson, D. and J.S. Jensen, *Mycoplasma genitalium: from Chrysalis to multicolored butterfly.* Clin Microbiol Rev, 2011. **24**(3): p. 498-514.

52. Workowski, K.A., G.A. Bolan, C. Centers for Disease, and Prevention, *Sexually transmitted diseases treatment guidelines, 2015.* MMWR Recomm Rep, 2015. **64**(RR-03): p. 1-137.

53. Mena, L.A., T.F. Mroczkowski, M. Nsuami, and D.H. Martin, *A randomized comparison of azithromycin and doxycycline for the treatment of Mycoplasma genitalium-positive urethritis in men.* Clin Infect Dis, 2009. **48**(12): p. 1649-54.

54. Schwebke, J.R., A. Rompalo, S. Taylor, A.C. Sena, D.H. Martin, L.M. Lopez, S. Lensing, and J.Y. Lee, *Re-evaluating the treatment of nongonococcal urethritis: emphasizing emerging pathogens--a randomized clinical trial.* Clin Infect Dis, 2011. **52**(2): p. 163-70.

55. Cho, I., S. Yamanishi, L. Cox, B.A. Methe, J. Zavadil, K. Li, Z. Gao, D. Mahana, K. Raju, I. Teitler, H. Li, A.V. Alekseyenko, and M.J. Blaser, *Antibiotics in early life alter the murine colonic microbiome and adiposity.* Nature, 2012. **488**(7413): p. 621-6.

56. Angelakis, E., M. Million, S. Kankoe, J.C. Lagier, F. Armougom, R. Giorgi, and D. Raoult, *Abnormal weight gain and gut microbiota modifications are side effects of long-term doxycycline and hydroxychloroquine treatment.* Antimicrob Agents Chemother, 2014. **58**(6): p. 3342-7.

57. Lane, J.A., L.J. Murray, I.M. Harvey, J.L. Donovan, P. Nair, and R.F. Harvey, *Randomised clinical trial: Helicobacter pylori eradication is associated with a significantly increased body mass index in a placebo-controlled study.* Aliment Pharmacol Ther, 2011. **33**(8): p. 922-9.

58. Kamada, T., J. Hata, H. Kusunoki, M. Ito, S. Tanaka, Y. Kawamura, K. Chayama, and K. Haruma, *Eradication of Helicobacter pylori increases the incidence of hyperlipidaemia and obesity in peptic ulcer patients.* Dig Liver Dis, 2005. **37**(1): p. 39-43.

59. Angelakis, E., N. Armstrong, C. Nappez, M. Richez, E. Chabriere, and D. Raoult, *Doxycycline assay hair samples for testing long-term compliance treatment.* Journal of Infection, 2015. **71**(5): p. 511-517.

60. Geisler, W.M., W.D. Koltun, N. Abdelsayed, J. Burigo, L. Mena, S.N. Taylor, B.E. Batteiger, A.R. Thurman, I.I.I.E.W. Hook, T.A. Vaughn, M.P. Annett, R.A. Muenzen, and J. Caminis, *Safety and Efficacy of WC2031 Versus Vibramycin for the Treatment of Uncomplicated Urogenital Chlamydia trachomatis Infection: A Randomized, Double-blind, Double-Dummy, Active-Controlled, Multicenter Trial.* Clinical Infectious Diseases, 2012. **55**(1): p. 82-88.

61. FDA. *505(b)(2) Efficacy Supplement, Resubmission/Class 2 Doxycycline Hyclate DR* 4/1/2019]; Available from: https://www.fda.gov/media/86171/download.

62. Lexicomp, *Doxycycline (Lexi-Drugs)* 2019.

63. Mayne. *Doxycycline Prescribing information* 1/5/2019]; Available from: https://dailymed.nlm.nih.gov/dailymed/getFile.cfm?setid=54ce5887-5e72-4d7a-a821-936dd3ffa68c&type=pdf&name=54ce5887-5e72-4d7a-a821-936dd3ffa68c

64. Jia, B., A.R. Raphenya, B. Alcock, N. Waglechner, P. Guo, K.K. Tsang, B.A. Lago, B.M. Dave, S. Pereira, A.N. Sharma, S. Doshi, M. Courtot, R. Lo, L.E. Williams, J.G. Frye, T. Elsayegh, D. Sardar, E.L. Westman, A.C. Pawlowski, T.A. Johnson, F.S. Brinkman, G.D. Wright, and A.G. McArthur, *CARD 2017: expansion and model-centric curation of the comprehensive antibiotic resistance database.* Nucleic Acids Res, 2017. **45**(D1): p. D566-D573.
