# Supplementary material for: Impact of doxycycline post-exposure prophylaxis for sexually transmitted infections on the gut microbiome and antimicrobial resistome
Source: Nat Med. 2024 Oct 3;31(1):207–17. doi: 10.1038/s41591-024-03274-2 (PMC11750720; doi:10.1038/s41591-024-03274-2)
Supplement: Supplementary file 2 — Reporting Summary [file 41591_2024_3274_MOESM2_ESM.pdf]

nature portfolio

Corresponding author(s): Charles R. Langelier

Last updated by author(s): Apr 12, 2024

Reporting Summary

Nature Portfolio wishes to improve the reproducibility of the work that we publish. This form provides structure for consistency and transparency in reporting. For further information on Nature Portfolio policies, see our Editorial Policies and the Editorial Policy Checklist.

Statistics

For all statistical analyses, confirm that the following items are present in the figure legend, table legend, main text, or Methods section.

n/a	Confirmed	
☐	☒	The exact sample size (*n*) for each experimental group/condition, given as a discrete number and unit of measurement
☐	☒	A statement on whether measurements were taken from distinct samples or whether the same sample was measured repeatedly
☐	☒	The statistical test(s) used AND whether they are one- or two-sided<br>*Only common tests should be described solely by name; describe more complex techniques in the Methods section.*
☐	☒	A description of all covariates tested
☐	☒	A description of any assumptions or corrections, such as tests of normality and adjustment for multiple comparisons
☐	☒	A full description of the statistical parameters including central tendency (e.g. means) or other basic estimates (e.g. regression coefficient) AND variation (e.g. standard deviation) or associated estimates of uncertainty (e.g. confidence intervals)
☐	☒	For null hypothesis testing, the test statistic (e.g. *F*, *t*, *r*) with confidence intervals, effect sizes, degrees of freedom and *P* value noted<br>*Give P values as exact values whenever suitable.*
☒	☐	For Bayesian analysis, information on the choice of priors and Markov chain Monte Carlo settings
☒	☐	For hierarchical and complex designs, identification of the appropriate level for tests and full reporting of outcomes
☐	☒	Estimates of effect sizes (e.g. Cohen's *d*, Pearson's *r*), indicating how they were calculated

Our web collection on statistics for biologists contains articles on many of the points above.

Software and code

Policy information about availability of computer code

Data collection	Participant clinical and demographic data , and the self-collected rectal swabs at enrollment and month 6 were obtained from the DoxyPEP randomized controlled trial (ClinicalTrials.gov registration number: NCT03980223). RedCAP (hosted at University of Washington) and Microsoft Excel (Raleigh, NC) were used for data collection for the DoxyPEP trial. All swabs with adequate DNA and RNA yield from extraction underwent DNA-sequencing and RNA-sequencing. All code can be found at https://github.com/infectiousdisease-langelier-lab/doxyPEP.
Data analysis	For microbial and antimicrobial resistance gene analyses, raw sequencing reads underwent quality filtration, removal of human reads and were input into the CZ ID pipeline (mNGS pipeline v8.1), which performs reference-based taxonomic alignment at both the nucleotide and amino acid level against sequences in the National Center for Biotechnology Information (NCBI) nucleotide (NT) and non-redundant (NR) databases, followed by assembly of the reads matching each taxon detected. Through the CZ ID AMR pipeline (v1.2.15), the reads were aligned against sequences from the CARD databases (canonical CARD v3.2.6 and WildCARD v4.0.0) to identify antimicrobial resistance genes.<br><br>Statistical analysis was performed in RStudio, version 2023.09.1+494 using R v4.2.1. Alpha and beta diversity calculations were performed using vegan v2.6.4. Bulk RNA-seq differential expression analysis was performed using DESeq2 package (v1.36.0). Figures were made using the ggplot2 (v3.5.1) packages for R.

For manuscripts utilizing custom algorithms or software that are central to the research but not yet described in published literature, software must be made available to editors and reviewers. We strongly encourage code deposition in a community repository (e.g. GitHub). See the Nature Portfolio guidelines for submitting code & software for further information.

Data

Policy information about availability of data

All manuscripts must include a data availability statement. This statement should provide the following information, where applicable:

- Accession codes, unique identifiers, or web links for publicly available datasets
- A description of any restrictions on data availability
- For clinical datasets or third party data, please ensure that the statement adheres to our policy

FASTQ files containing non-host reads identified by the CZ ID pipeline, following subtraction of reads aligning to the human genome, are available from the National Center for Biotechnology Information (NCBI) Sequence Read Archive (SRA) under BioProject ID PRJNA1099775. All datasets and source data can be found at https://github.com/infectiousdisease-langelier-lab/doxyPEP.

Research involving human participants, their data, or biological material

Policy information about studies with human participants or human data. See also policy information about sex, gender (identity/presentation), and sexual orientation and race, ethnicity and racism.

Reporting on sex and gender

All participants were assigned male sex at birth in the DoxyPEP trial as doxy-PEP is primarily being studied as an intervention in the population of men who have sex with men or transgender women Therefore, we did not perform any subanalyses based on sex or gender. Within the DoxyPEP trial, 96% of participants were male gender, and 4% were transgender women.

Reporting on race, ethnicity, or other socially relevant groupings

The race variable and the ethnicity variable were combined and re-categorized as a single variable "race/ethnicity." Categories were "non-Hispanic White" (49%), "Hispanic White" (17%), "Asian" (11%), "Black or African American" (4%), "Other or Multiracial" (15%), and "Missing" (3%). Categories were created based on self-reported race and ethnicity data. The race/ethnicity distribution was similar between the standard of care arm and the doxy-PEP arm. We did not use race/ethnicity as a proxy for any other variables (e.g., socioeconomic status). Confounding variables such HIV infection status (living with HIV or on HIV pre-exposure prophylaxis) was accounted for in linear regression model as a sensitivity analysis.

Population characteristics

Of the participants with analyzable DNA-seq samples, participants were a median age of 38 years (IQR: 32-50 years). Almost half (49%) were non-Hispanic White, followed by Hispanic White (17%), Asian/Pacific Islander (11%), Black/African American (4%). Among the participants, 39% were living with HIV and 61% were on HIV PrEP. Almost all (98%) had a stable living situation. For the primary outcome of proportional mass of ARG class to the resistome, we used linear regression models to account for the HIV infection status as a covariate.

Recruitment

The study was conducted at two HIV clinics and two sexual health clinics in San Francisco and Seattle. Participants were eligible if they were at least 18 years of age, were assigned male sex at birth, had received a diagnosis of HIV or were taking or planning to start HIV PrEP, had a history of condomless anal or oral sex with a man in the previous 12 months, and had received a diagnosis of gonorrhea, chlamydia, or early syphilis in the previous 12 months. Participants were not eligible if they reported a tetracycline allergy, were taking medications with drug interactions with doxycycline, or were planning to take doxycycline for an extended period.

Ethics oversight

The study protocol was approved by the University of California, San Francisco institutional review board, which served as the primary institutional review board.

Note that full information on the approval of the study protocol must also be provided in the manuscript.

Field-specific reporting

Please select the one below that is the best fit for your research. If you are not sure, read the appropriate sections before making your selection.

☒ Life sciences ☐ Behavioural & social sciences ☐ Ecological, evolutionary & environmental sciences

For a reference copy of the document with all sections, see nature.com/documents/nr-reporting-summary-flat.pdf

Life sciences study design

All studies must disclose on these points even when the disclosure is negative.

Sample size

No sample size calculation was preformed as we leveraged this analysis from a randomized controlled trial of Doxy-PEP use (DoxyPEP trial, ClinicalTrials.gov registration number: NCT03980223). To our knowledge, this is the largest microbiome/resistome study (n=99 individual participants from both DNA- and RNA-seq data) evaluating changes in the antimicrobial resistance genes found in the gut resistome over a 6 month follow-up period with a comparison control group. Many microbiome and resistome studies have a much smaller population size (n <50), and our sample size was robust enough for significant findings.

Data exclusions

Water samples were processed in parallel with the participant samples, and we used previously described negative binomial model to exclude microbes and antimicrobial resistance genes likely to be contaminants from the laboratory environment. We also excluded DNA-seq samples that had low complexity (duplicate compression ratio>=10) and <100,000 reads sequenced. Antimicrobial resistance genes with a <5% read coverage breadth were excluded from analysis.

Replication	No external datasets were available for replication.
Randomization	Participants in the DoxyPEP trial were randomized 2:1 to the intervention (doxy-PEP use) vs control (standard of care). In the DoxyPEP trial, randomization was performed according to study clinic with the use of variable block size and was stratified according to site.<br><br>For this analysis, a subset of 150 participants from the 510 DoxyPEP trial participants was selected for metagenomic sequencing of self-collected rectal swab samples. The 150 participants were selected based on the following criteria: 1) study arm group (50 SOC, 100 doxy-PEP), 2) HIV infection status (1:1 of participants living with HIV and participants on HIV PrEP), and 3) availability of both day-0 and month-6 rectal samples. The SOC participants were a simple random sample, while the doxy-PEP participants were the top 50 participants, including both persons with and without HIV infection, with the highest reported combined doxy-PEP use on the month-3 and month-6 study visits.
Blinding	Participants of the DoxyPEP trial were not blinded as the study was meant to evaluate the net effectiveness of the intervention, including biologic efficacy and potential changes in sexual behavior with doxy-PEP use.

Reporting for specific materials, systems and methods

We require information from authors about some types of materials, experimental systems and methods used in many studies. Here, indicate whether each material, system or method listed is relevant to your study. If you are not sure if a list item applies to your research, read the appropriate section before selecting a response.

Materials & experimental systems

n/a	Involved in the study
☒	☐ Antibodies
☒	☐ Eukaryotic cell lines
☒	☐ Palaeontology and archaeology
☒	☐ Animals and other organisms
☐	☒ Clinical data
☒	☐ Dual use research of concern
☒	☐ Plants

Methods

n/a	Involved in the study
☒	☐ ChIP-seq
☒	☐ Flow cytometry
☒	☐ MRI-based neuroimaging

Clinical data

Policy information about clinical studies

All manuscripts should comply with the ICMJE guidelines for publication of clinical research and a completed CONSORT checklist must be included with all submissions.

Clinical trial registration	NCT03980223
Study protocol	Study protocol is available in the Supplementary Information pdf file.
Data collection	The study was conducted at two HIV clinics and two sexual health clinics in San Francisco and Seattle from August 19, 2020, through May 13, 2022.
Outcomes	Outcomes and CONSORT checklist not applicable, as this study was not a clinical trial (only used data generated from the clinical trial).

Plants

Seed stocks	*Report on the source of all seed stocks or other plant material used. If applicable, state the seed stock centre and catalogue number. If plant specimens were collected from the field, describe the collection location, date and sampling procedures.*
Novel plant genotypes	*Describe the methods by which all novel plant genotypes were produced. This includes those generated by transgenic approaches, gene editing, chemical/radiation-based mutagenesis and hybridization. For transgenic lines, describe the transformation method, the number of independent lines analyzed and the generation upon which experiments were performed. For gene-edited lines, describe the editor used, the endogenous sequence targeted for editing, the targeting guide RNA sequence (if applicable) and how the editor was applied.*
Authentication	*Describe any authentication procedures for each seed stock used or novel genotype generated. Describe any experiments used to assess the effect of a mutation and, where applicable, how potential secondary effects (e.g. second site T-DNA insertions, mosiacism, off-target gene editing) were examined.*
